# Movers and Stayers: A Study of Emigration from Sweden 1993–2014

**DOI:** 10.1007/s10680-022-09634-3

**Published:** 2022-08-30

**Authors:** Debora Pricila Birgier, Christer Lundh, Yitchak Haberfeld, Erik Elldér

**Affiliations:** 1 The Department of Economy and Society, University of Gothenburg, Gothenburg, Sweden; 2 Centre for Economic Demography, Lund University, Lund, Sweden; 3 Department of Labor Studies, Tel Aviv University, Tel Aviv, Israel

**Keywords:** Emigration, Self-selection, Unobserved attributes, Sweden

## Abstract

A standard proposition in the migration literature is that emigrants are not drawn randomly from their source population, but rather compose a self-selected group in terms of labour market characteristics. Such self-selection refers to observed characteristics, such as education, or occupation, as well as unobserved characteristics such as cognitive abilities. However, due to data limitations, most previous studies on selectivity have analysed immigrants’ characteristics at destinations rather than using data from their source countries. This paper assesses emigrants’ selectivity patterns by following the full-risk population of natives over a long period of time (over 20 years). It also includes an innovative measure of selectivity on unobserved characteristics—namely, school performance—as a proxy for individual motivation and cognitive abilities, and it compares it to the widely used measure of income residuals. We use Swedish register data and assess the probabilities of leaving Sweden between 1993 and 2014 among men and women born in Sweden between 1975 and 1978. We further look for differences among Swedish emigrants who chose different countries of destination. The findings suggest that emigrants are positively self-selected in terms of their observed characteristics, whereas selectivity patterns in terms of unobserved characteristics are more complex. When we assess unobservable characteristics using compulsory school grades as a proxy, emigrants are found to be positively self-selected, while when using income residuals, we find that the effect is U-shaped. Individuals leaving to non-Nordic countries are also found to be more positively self-selected than those heading to neighbouring countries. We discuss these findings and their implications in light of economic and sociological theories.

**Supplementary Information:**

The online version contains supplementary material available at 10.1007/s10680-022-09634-3.

## Introduction

The decision to emigrate from a country is shaped by various micro-, macro- and meso-levels factors (Hammar & Tamas, 1997). This paper takes a micro-level perspective using an economic approach to explain migration decision making (Borjas, 1994; Roy, 1951). According to this perspective, the decision about whether or not to emigrate is based on the relative returns on skills in the source as well as in the destination countries. Patterns of selective out-migration (henceforth emigration) indicate how potential emigrants estimate returns to their skills in the labour market of their home country compared to their potential returns in possible countries of destination. The economic model of migration also asserts that within the population of emigrants, the most intensive positive self-selection patterns (“sorting”) are found among the most highly skilled individuals, who choose to move to highly developed countries with free markets and with restrictive welfare policies (Grogger & Hanson, 2011). Due to data limitations, most previous studies on selectivity have analysed immigrants’ characteristics at destinations,^1^ and only recently have scattered studies explored the selectivity patterns of emigrants at their source countries (Borjas et al., 2019; Gould & Moav, 2016; Rosso, 2019). However, only a few of those studies have considered the entire native population-at-risk and followed individuals over a relatively long period of time (for an exception, see Borjas et al., 2019). In addition, very few studies have included information on individual attributes that can serve as efficient proxies of unobserved individual abilities (for exceptions, see Gibson & Mckenzie, 2011; Maleszyk, 2021).

Furthermore, a vast share of the migration literature focuses on the migration from developing to developed countries. However, migration between two developed countries constitutes about one fifth of all international migration. As of 2000, 28 million migrants have moved between developed countries, half of whom were highly skilled (Artuc et al., 2015; Özden et al., 2011). By adjusting their migration policies, countries compete to attract high-skilled immigrants (Commander et al., 2004; Iredale, 1999; Mahroum, 2001; Ouaked, 2002). Therefore, the present paper systematically assesses the patterns of selectivity in emigration to multiple destinations from a highly developed country (Sweden) based on rich longitudinal information on the entire population of natives.

The overarching aim of the present study is to test the dominant selectivity hypothesis, and its contribution is two-fold. First, we use data that enable testing of emigrants’ positive self-selection through a long-term, pre-migration follow-up of the entire population at risk, focusing on the Swedish birth cohorts of 1975–1978, and by examining selectivity and sorting patterns to different destinations over 22 years (1993–2014). Second, we use an innovative perspective to assess selectivity patterns by looking at pre-market performance in compulsory education as a proxy for individuals’ motivation and cognitive abilities. This proxy is compared to the commonly used labour market performance-proxies’ indicators. We estimate individuals’ probabilities of emigrating during the study period and their choice of destination, grouped into four main destination categories.

## Theory and Previous Studies

### The Roy Model

As stated, while the decision to emigrate is shaped by different micro-, macro- and meso-level factors (Hammar & Tamas, 1997), the current paper takes a micro-level perspective using an economic approach to migration decision making.^2^ Within this decision making framework, the rational choice theory sees migration as an individual investment decision, aimed at maximizing the net benefit from a set of alternatives (Haug, 2008). This decision entails risks and costs that immigrants choose to engage in for improving their future economic well-being at their destination. Migration occurs when immigrants’ expected net return on their investment in migration is positive. The original argument made by Chiswick (1978, 1999) suggested that individuals with higher skill levels believe that their investment in migration will pay off, and they are therefore more likely to migrate. Positively self-selected emigrants are generally described as more abled and ambitious than individuals who choose to remain in their place of origin. This positive self-selection refers both to observed characteristics, such as education, work experience and occupation, as well as to unobserved characteristics, such as motivation, risk-taking behaviours, destination-language proficiency, and cognitive abilities (Chiswick, 1978).

However, neither the between-country differences in mean incomes nor migration costs solely determine the type of selection that characterizes emigration flows. Borjas (1987), in his seminal paper, applied the Roy model (1951) to the migration framework and argued that the decision to migrate is also based on the relative level of returns to skills between origin and potential countries of destination. Borjas suggested that these differences in returns can be estimated through the difference between the levels of income inequality in source and host countries, and that immigrants are selected both positively and negatively as a function of between-country differences in income inequality. Positive selection occurs when the host country provides higher returns for skills, as manifested by a higher level of income inequality at destination compared to the source country, and negative self-selection takes place when the incomes distribution in the source country has a larger variance than that of the destination country (Borjas, 1994). In the same vein, sorting refers to the continuation of the initial selection process of leaving the country of origin, in which immigrants from the same source country differ in their destination choices, based on differences in income inequality at potential destinations. Positive sorting implies that the most positive self-selected individuals within the emigrant population are attracted to countries with highest returns to skills (Grogger & Hanson, 2011).

Therefore, the Roy model predicts different types of selectivity on observed and unobserved earnings-related attributes, depending on the relative gaps in returns to observed and unobserved characteristics between the countries of origin and destination. Empirically, previous studies have reached conflicting results on selectivity levels when looking at both education (a measure of observed attributes) and at emigrants’ residual wages (a proxy for unobserved characteristics). In line with the Roy model, Borjas et al. (2019) found positive selectivity in terms of earnings and earning residuals in the case of emigrants from Denmark, a country with low levels of inequality. They showed that most of the positive self-selection is due to unobserved characteristics^3^ arguing that looking solely at educational levels undermines a large share of emigrants’ selectivity. However, other studies have found intermediate selection, showing that emigrants are drawn from the middle of the wage distribution, as in the case of emigrants from Mexico and Israel to the US (Chiquiar & Hanson, 2005; Gould & Moav, 2016), while others found negative patterns of self-selection among emigrants (Moraga, 2011). Such mixed results confirm the model’s expectation that self-selection patterns are sensitive to the specific source and destination countries under observation (Dustmann & Görlach, 2015; Parey et al., 2017; Rosso, 2019).^4^ However, even when using the same source and destination countries, as in the case of Mexican emigrants to the US, findings are inconsistent for patterns of self-selection (Chiquiar & Hanson, 2005; Kaestner & Malamud, 2014; Moraga, 2011). Educational-occupational mismatch at origin on the one hand, and job match on the other have been suggested as a plausible explanation for the inconsistencies in findings regarding the relationship between education and emigration (Quinn & Rubb, 2005). This explanation might be relevant also in the case of earnings residuals, so that individuals with low job match are more prone to migrate (Villarreal, 2016).

Obviously, these studies restricted their samples to individuals with high levels of labour market attachment (by restricting samples’ ages, working hours, and earnings before migration) in order to identify their earning potential. However, a large share of emigration events occurs at early ages, shortly after graduating from high school or college, and therefore before individuals fully display their labour market potential (Kaestner & Malamud, 2014; Nekby, 2006). Thus, restricting the sample by age or labour market attachment levels implies that the research focuses on the right tail of the earnings distribution. Consequently, it might yield biased results for emigrants’ self-selection patterns.^5^

To address this issues, we look at alternative measures of abilities. For example, Schmidt and his colleagues recently used one possible proxy for ability by looking at the relative position of individuals on the educational distribution of their country of origin as an overall proxy for unmeasured characteristics as motivation, skills and resources (Schmidt et al., 2021). They show that, on average, emigrants are positively selected in terms of their relative education (compared with the distribution of stayers). An alternative possibility is to look for direct measures of abilities such as cognitive and non-cognitive tests. Previous studies that used tests of cognitive and non-cognitive abilities have found that such tests can explain a variety of life outcomes, such as educational choices, employment, occupation, earnings and other behaviours that involve risks (Heckman et al., 2006). Unfortunately, we do not have a direct measure of cognitive and non-cognitive abilities available to us in our data.^6^ Instead, we use individual average grades in ninth-grade, which is the last year of compulsory education in Sweden.^7^ Clearly, this measure is far from being a perfect proxy for individual abilities, both observed and unobserved. However, to the extent that individuals’ motivation and aptitudes are assessed by standardized tests, we believe that they can serve as a proxy for a set of abilities and motivations.^8^

In a recent paper, Maleszyk (2021) used a survey that included information on school exam results to assess migrants’ youth selectivity levels from a peripheral region in Poland. He showed that youths’ international migration could be described as having a U-shaped selectivity pattern in terms of their exam grades. The U-shaped pattern was explained by positive self-selection to continue higher education abroad by the most talented individuals and negative self-selection due to economic migration of those with low grades, who are more prone to move overseas to seek employment (Maleszyk, 2021).

By using such alternative proxies for individual achievements, which are probably related to unobservable attributes that are highly relevant to labour market success, we can also shed light on self-selection patterns among young emigrants that are still lacking indicators of actual market outcomes. In so doing we do not argue that grades are superior to income residuals, but rather introduce another measure of unobserved abilities, which is applicable to a larger share of the populations. This is even more important when taking into account the inconsistencies in the literature regarding the selection on income residuals. Using both measures is expected to enrich the empirical estimates in the literature.

### Gender and Diversity

Efforts have been made to include gender and other diversity dimensions in the general models of self-selection. Starting with gender, studies of international migration indicate a rise in female migration, not only among low-skilled women but also among the high skilled. It was found that skilled women have a higher propensity to migrate than skilled men (Docquier et al., 2012). The economic theory assumes that male migration is driven by economic factors, while female migration is also related to family constraints, thus arguing that many migrant women can be seen as “tied movers” (Bielby & Bielby, 1992; Borjas & Bronars, 1991; Docquier et al., 2012; Mincer, 1978). Such arguments imply that the selectivity patterns for women are less intense than those among men. Indeed, previous studies have presented evidence that women tend to be less positively self-selected relative to their male counterparts (Borjas et al., 2019; Junge et al., 2014). This paper therefore examines whether the self-selection mechanisms we hypothesize are similar among both women and men.

While this paper focuses on emigration of natives only, some natives have one or two foreign-born parents (i.e. are second-generation immigrants). It might be possible that having a family background of migration is associated with higher probabilities of emigrating, independently of individual attributes. In order to take into account possible effects of gender and parents’ country of birth, we classified the individuals by gender and we controlled for the birthplace of their parents (Sweden vs. other countries).

### The Swedish Case Study

Most studies that examine the Roy model of natives’ emigration from Scandinavian countries used mainly historical data focusing on the great emigration to the US at the end of the nineteenth century and the beginning of the twentieth century (from Norway (Abramitzky et al., 2012) and Sweden (Dribe et al., 2021)). These studies find that migrants to the US were negatively self-selected in the case of Norwegians, conforming to the expectations of the Roy model and middle levels of selectivity in the case of Sweden.^9^ Studies using recent data that assess the emigration pattern from Sweden (Nekby, 2006) and Denmark (Borjas et al., 2019) during the 1990s and the 2000s show that migrants are positively selected in terms of education. Using the contemporary Scandinavian countries as the source of emigration has two main benefits—first, the longitudinal register data quality, which contains information about emigration destination. Second, the lower levels of inequality relative to almost all other destinations (except other Scandinavian countries) make it easy to model the prediction of the Roy model (Borjas et al., 2019).

Based on the Roy model and concerning the cross-country levels of inequality and migration costs, we can expect that emigrants from Sweden will be positively self-selected due to the low levels of inequality in Sweden. Nonetheless, the rising levels of inequality in Sweden during the studied period^10^ might also promote negative self-selection. At the same time, some differences in the intensity of the selection might exist due to sorting to various destinations. We expect the most intense positive self-selection to distant countries with a free market, such as North America and Australia, compared to emigrating to a neighbouring Nordic country.^11^ Also, moving to another country in Europe is legally simpler than moving to North America but more complex than moving to a neighbouring Nordic country. When we compare the Nordic countries during the period in terms of inequality, Denmark had the lowest level during 1998–2010, followed by Finland, Norway and then Sweden (Parey et al., 2017). In addition, while all Nordic countries were suffering from an economic crisis during the 1990s, the unemployment rate in Sweden was higher (7.9) than that in Denmark (7.1), Norway (4.9) and Iceland (3.7). The only exception was Finland, with an unemployment rate higher than in Sweden of 13.4 (authors’ computation, based on ILO [2020] data). In our analysis, we first assess the self-selection of migrants in general. Then we present results for four global destinations: (1) North America and Oceania; (2) Nordic countries; (3) Western and Southern Europe; and (4) Other destinations. Because the number of emigrants is relatively small, we could not divide the destination countries into, more detailed regions.^12^ Between 1993 and 2014, about 3.4 to 5.5 per cent of the Swedish population left Sweden each year (Statistics Sweden, 2021).

It should be noted that it is possible for Swedish residents to emigrate without registering, suggesting that even when considering the high quality of the register data, some measurement errors might exist. Related to this, in recent years there is a growing attention to the concept of over-coverage of register data, referring to cases in which individual are registered as living in a country and in practice they left it (Monti et al., 2020; Wallace & Wilson, 2022). While these studies suggest that the extent of over-coverage is more substantial among migrants (Monti et al., 2020; Wallace & Wilson, 2022), they also show that there are variation along people's life course, with higher tendency of over-coverage at early ages (Monti et al., 2020).^13^ However, we expect that the number of unregistered migration events would be small because it is a legal requirement to report emigration, and because emigration has tax implications.^14^ Nonetheless, it is not uncommon for people to leave Sweden and report it only retrospectively. In these cases, the date of emigration is recorded as the date on which the authorities receive notification of emigration (Nekby, 2006). This delayed registration can result at lower earnings and incomes of emigrants to continue receiving transfer payments or return to Sweden as well as leaving at the middle of the calendar year, in practice affecting our estimations of the relation between income residuals and emigration.

## Study Design and Method

### Data

The data used in this study were obtained from GILDA,^15^ which includes longitudinal individual-level data from the Swedish registers held by Statistics Sweden (SCB) for the years 1990–2014, covering the entire Swedish population. The information on emigration is taken from the migration registers for the years 1990–2014. The migration registers report the date of emigration and the emigrant’s country of destination.

### Population

The population of this study includes men and women born in Sweden between 1975 and 1978, who were expected to complete their compulsory education between 1990 and 1993. We followed this cohort from the ages of 18–39 years old (1993–2014), and assessed their probability of leaving Sweden during this period. We chose to look at a birth cohort for which ninth-grade school tests were national and standardized. The school grading policy was uniform across Sweden between 1960 and 1996, and thereafter underwent a significant change (Wikström, 2006). The data at hand follow individuals who completed their ninth grade from 1990 only, thus restricting our ability to follow earlier cohorts. However, because the major migration ages are between 20 and 40 (Kaestner & Malamud, 2014; Nekby, 2006),^16^ focusing on this cohort makes it possible to view most of its emigration cases. In total, the population (henceforth the full sample) consisted of 188,159 individual men (corresponding to 3,763,205 person-year observations) and 178,905 individual women (corresponding to 3,554,384 person-year observations), for whom there was information on all relevant variables.^17^ In some sections, we used a restricted sample of individuals with labour market attachment in order to compare our results to those of previous studies.^18^ We were able to identify 8067 male and 10,173 female emigrants in our full sample.^19^

### Design and Models

We modelled the emigration probabilities of natives in two ways. First, we estimated a binary logit model on the transformed probability of emigrating versus not emigrating for native-born Swedes who lived in Sweden each year, where we followed natives from the age of 18 until they were censored (due to an emigration event, due to reaching the age of 39, or death).^20^ In this part, we estimated the model twice. First, we followed the entire population at risk: native-born individuals at the ages of 18–39, and then followed a restricted subpopulation composed of individuals with labour market attachment in the previous year.^21^ This was done for estimating unobserved abilities for those subpopulations by using their residual incomes, a procedure we cannot perform for those with no market attachment.

Second, we estimated a multinomial logit model on the transformed probability of emigrating to four different global regions. This model simultaneously estimated the impact of a set of explanatory variables on four different emigration outcomes—emigration to (1) the Nordic countries; (2) North America and Oceania; (3) Western and Southern European countries; and (4) the rest of the world. The base outcome of this multinomial model was staying in Sweden. These estimates indicated the differential effects of the explanatory variables on the different destination choices. The decision to divide immigration destinations in this way was based on our expectations of the Roy model and the fact that there are significant differences between emigration to these four different destinations as explained in the introduction. As explained above, we expected to have gender-based differences in both selection intensity and in the effects of the explanatory variables on the emigration outcomes estimated. Consequently, all models were estimated separately for men and women.

### Variables

The dependent variable in this study was emigrating from Sweden. It is defined as leaving Sweden for more than 36 consecutive months, which enables us to focus on long-term emigration, which is at the centre of the present study. The decision to define emigration as leaving for a period equivalent to, at least, three years, results from the reality that some individuals may choose to acquire education abroad, which can be seen as a transitory migration decision. We focused on the first emigration event of individuals after the age of 18, as they are defined in most of the literature as adult migrants.^22^ Individuals who left for a period longer than 12 months but shorter than 36 months are considered to have experienced a short migration event, which is represented by a dummy variable in the models.^23^ Individuals can have just one long-term emigration event at the time under observation and are censored after its occurrence.

Our main independent variables were designed to assess different aspects of self-selection among emigrants and included education level, ninth grade average exam scores and income residuals decile. Starting with educational level, this is a time-varying sequence of five dummies of highest educational levels achieved, in which the omitted category is completing secondary education (*gymnasium*—12th grade).^24^ Completing secondary education is not obligatory in Sweden (but rather nine years of education).^25^

The second measure, the ninth-grade scores are based on the national exams conducted during the last year of compulsory schooling *(högstadiet)*. They are based on the grading policy for the *högstadiet* level, and with the belief that the written and formal forms of testing should be as scientifically objective as possible (Widén, 2010; Wikström, 2006). The grading scheme assigned a value of 1 to 5 in each learning subject, and the final grade is the mean of these values. To facilitate the interpretation of this variable, we used the percentile of the grade that an individual achieved on the grades distribution of their graduation cohort (resulting in a variable ranging from 0 to 100). About 2 per cent of each birth cohort did not have a ninth grade score and they were omitted from our analysis.

The last measure of selectivity is the decile of an individual’s lag residual income derived from a standard Mincerian income model. This residual is assumed to represent a measure of the unobserved individual characteristics. Obviously, this measure could be derived only for the restricted sample of individuals with labour market attachment (define by being 25 or older and having positive lagged income). The income residual is based on an income model including age (and its squared term), education (measured by five dummies), marital status, having a child under the age of three, number of children, and fixed-effects of year and county dummies.^26^ We used two types of income to calculate these residuals. First, we followed previous studies and used gross income from work and self-employment. Second, income including work-related insurance benefits—we used a more inclusive variable that includes parental leave benefits, unemployment benefits, students’ allowances and illness or injury allowances. All of these benefits are supposed to be directly related to individuals’ previous incomes from work, and so using them in a sensitivity analysis enables us to include a substantially larger share of the emigration events.^27^ The income residuals are then divided into deciles—from decile 1 (containing the largest negative values) to decile 10 (the largest positive values) to show the emigration probabilities at different locations on the lag income residual distribution.^28^ This decile of residuals from the income models represents the effect of unobserved abilities on the probability of emigrating.

Finally, we controlled for several demographic variables, including age (and its squared term), marital status, having children, and parents’ country of birth. Marital status is based on the civil status in Swedish registers. The variables for the number of children and having a child under the age of three were derived from the household-level data in the Swedish registers. Finally, parents’ country of birth was an indicator variable with three categories: both parents were born in Sweden (the omitted category), one was born in Sweden and one abroad, and both parents were born abroad.

## Results

### Descriptive Overview

As stated above, the results were based on two samples: the full sample, which consists of all individuals at the ages of 18–39, and the restricted sample, which is composed of individuals with labour market attachment (defined by age, 25–39, and positive lag annual incomes). Appendices 1 and 2 present the descriptive statistics of the full sample and the restricted sample, respectively, by gender. Starting with the full sample, about four per cent of men and six per cent of women emigrated from Sweden during the period 1993–2014. Among men, most emigrants left either to Nordic countries or to Western and Southern Europe, while among women, the majority of emigrants left to Western and Southern Europe followed by Nordic countries, North America and Oceania. Among men, emigrants’ educational levels are higher than those of the stayers, whereas among women, the education levels of emigrants and stayers do not differ much. The average percentile of school grades for emigrant men is much higher (almost by 14 points) than that of stayers, and among women, the equivalent gap is about 10 points. This implies that, at least according to the descriptive statistics, emigrants have higher abilities than stayers.

When looking at the restricted sample (i.e. individuals with labour market attachment) as presented in appendix 2, we can see that the share of emigrants from their birth cohort is reduced to about three per cent. However, in the restricted sample, emigrants showed a more intensive pattern of positive self-selection in terms of education levels and school grades compared to emigrants in the entire sample. Specifically, when we restricted the sample to women with labour force attachment, emigrant women have higher educational levels compared to stayers.

On average, emigrants from Sweden had lower income relative to stayers. This can be explained by the younger age of emigrants compared to those staying in Sweden. Nonetheless, even when looking at the mean income of stayers when they were 29 to 30, their incomes (and income including work-related insurance benefits) are higher than that of emigrants. As stated previously, this might also be due to a delay in the registration of emigration as well as leaving at the middle of the calendar year. Emigrants tend to be located at a lower incomes-residuals decile relative to non-emigrants, both for men and for women. At the same time, however, men emigrants were also overrepresented at the highest decile. The difference between emigrants and stayers on the other proxy of unobserved attributes—their percentile in the school grades distribution—was higher in the restricted than in the full sample. The remaining control variables did not seem to differ much between the full sample and the restricted sample. In sum, the descriptive statistics suggest that emigrants were positively self-selected from their birth cohort in terms of education and school performance, but there were gender (and destination-based) differences in the intensity levels within this general pattern of emigrants’ positive self-selection.

### Selectivity on Observed Characteristics—Educational Levels

**Fig. 1:**
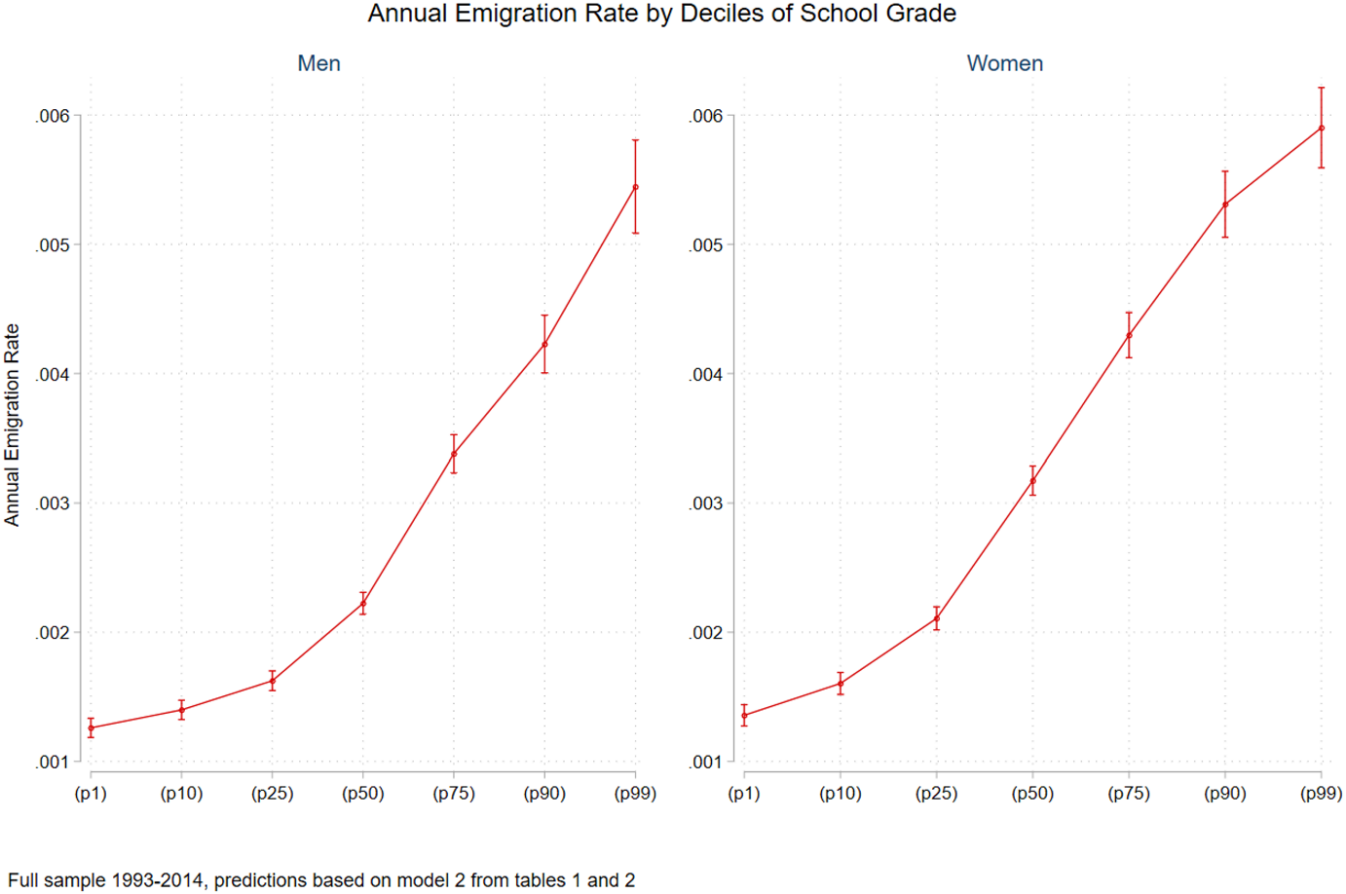
Annual emigration rate by deciles of school grade, men and women

**Fig. 2:**
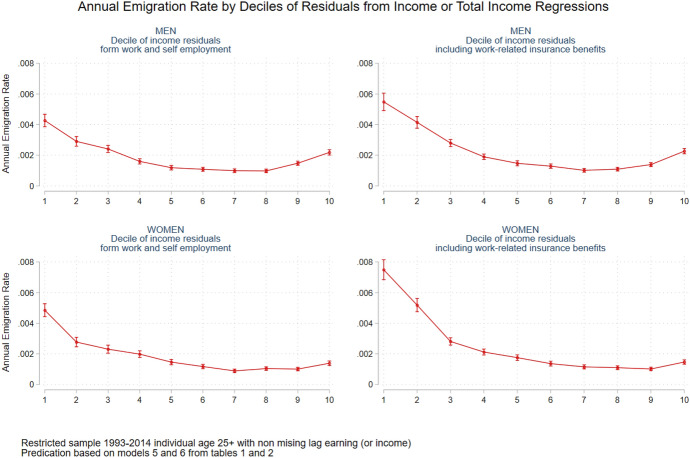
Annual emigration rate by deciles of residuals from income or total income regression, men and women

Tables 1 and 2 present the results from the logistic models predicting the probability of emigrating from Sweden for the full sample and for the restricted sample of men and women, respectively. Figures 1 and 2 present the predicted yearly emigration rate based on different predictors derived from the estimated models. As can be seen from Model 1 in Table 1, the effect of educational level on the odds ratios to emigrate from Sweden is U-shaped among men. Individuals with nine years of education or less have a higher probability of emigrating relative to individuals with secondary education, and the same is true for those having higher education. The effect of higher education on emigration is stronger as education levels rise. Once we control for school grades (Model 2 in Table 1), the effect of having post-secondary non-academic education becomes negative, while the rest of the effects remain in the same direction. Nevertheless, the effect of higher education is reduced substantially, while the effect of compulsory education increases. Among women, having a postgraduate education is associated with a higher probability of emigrating, whereas having higher education that is not postgraduate is associated with a lower probability relative to women with secondary education (Models 1 and 2 in Table 2). For women, controlling for school performance diminishes the effect of postgraduate education and the effect of low education levels becomes positive. Overall, it seems that the tendency to leave Sweden based on educational levels is highly dependent on abilities as assessed by school performance.

**Table 1:** Logistical models for predicting emigration from Sweden 1993–2014: individual men born in Sweden between 1975 and 1978 (OR)

	Full sample		Restricted sample			
	(1)	(2)	Age 25+ (3)	Age 25+ and lagged income (4)	Age 25+ and lagged income and benefits^1^	
					(5)	(6)
*Educational level—omitted—secondary education*						
Compulsory education 9 years or less	1.099* (0.045)	1.385* *(0.058)	1.465** (0.080)	1.498** (0.120)	1.307** (0.105)	1.126 (0.087)
Post-secondary education less than two	1.221** (0.058)	0.857** (0.042)	1.019 (0.060)	1.116 (0.090)	1.013 (0.081)	1.055 (0.074)
Higher education two years or longer	2.073** (0.058)	1.251** (0.041)	1.205** (0.044)	1.830** (0.086)	1.663** (0.079)	1.485** (0.067)
Postgraduate	6.480** (0.521)	3.089** (0.261)	2.856** (0.245)	5.067** (0.509)	5.125** (0.516)	4.501** (0.443)
CSN^2^	1.046 (0.036)	1.009 (0.036)	1.191** (0.049)	1.239** (0.064)	0.782** (0.045)	1.083 (0.053)
Percentile of school grade		1.015** (0.001)	1.018** (0.001)	1.015** (0.001)	1.015** (0.001)	1.016** (0.001)
*Decile of income residual—omitted 1st decile*						
2nd decile					0.679** (0.046)	0.754** (0.053)
3rd decile					0.563** (0.037)	0.507** (0.034)
4th decile					0.373** (0.028)	0.343** (0.025)
5th decile					0.276** (0.022)	0.266** (0.021)
6th decile					0.252** (0.020)	0.232** (0.018)
7th decile					0.230** (0.018)	0.183** (0.015)
8th decile					0.227** (0.018)	0.197** (0.015)
9th decile					0.344** (0.024)	0.251** (0.018)
10th decile					0.510** (0.033)	0.410** (0.027)
Constant	0.000** (0.000)	0.000** (0.000)	0.000** (0.000)	0.000** (0.000)	0.000** (0.000)	0.000** (0.000)
Observations (individual*year)	3,763,205	3,763,205	2,452,999	2,277,727	2,277,727	2,337,644
Individuals	188,159	188,159	185,526	183,098	183,098	184,151
Emigration events	8067	8067	6237	3858	3858	4397

All models control for age (and its square term), marital status, number of children, and having a child under the age of tree, migration background, and previous short migration event. Omitted category in the models—those who did not emigrate

Individual clustered robust standard errors in parentheses

^1^Income including work-related insurance benefits

^2^An indicator of receiving educational allowances

**p < 0.01; *p < 0.05

**Table 2:** Logistical models for predicting emigration from Sweden 1993–2014: Individual women born in Sweden between 1975 and 1978 (OR)

	Full sample		Restricted sample			
	(1)	(2)	Age 25+ (3)	Age 25+ and lag income (4)	Age 25+ and lagged income and benefits^1^	
					(5)	(6)
*Educational level—omitted—secondary education*						
Compulsory education 9 years or less	0.871** (0.033)	1.101* (0.043)	1.351** (0.079)	1.479** (0.148)	1.291* (0.130)	1.068 (0.092)
Post-secondary education less than two	0.933 (0.035)	0.696** (0.026)	0.848** (0.041)	1.115 (0.084)	1.082 (0.081)	0.943 (0.059)
Higher education two years or longer	0.865** (0.021)	0.560** (0.016)	0.523** (0.017)	1.087 (0.053)	1.036 (0.051)	0.776** (0.034)
Postgraduate	2.438** (0.261)	1.231 (0.134)	1.077 (0.118)	3.121** (0.413)	3.036** (0.403)	2.192** (0.275)
CSN^2^	0.913** (0.026)	0.866** (0.025)	1.025 (0.036)	1.111* (0.054)	0.665** (0.036)	1.028 (0.045)
Short migration	3.809** (0.156)	3.542** (0.147)	3.195** (0.147)	3.135** (0.228)	2.797** (0.205)	2.780** (0.176)
Percentile of school grade		1.015** (0.000)	1.020** (0.001)	1.015** (0.001)	1.016** (0.001)	1.018** (0.001)
*Decile of income residual—omitted 1st decile*						
2nd decile					0.569** (0.039)	0.689** (0.042)
3rd decile					0.473** (0.033)	0.371** (0.023)
4th decile					0.406** (0.029)	0.280** (0.018)
5th decile					0.299** (0.023)	0.231** (0.016)
6th decile					0.240** (0.019)	0.180** (0.013)
7th decile					0.182** (0.015)	0.152** (0.011)
8th decile					0.212** (0.016)	0.145** (0.010)
9th decile					0.206** (0.015)	0.134** (0.010)
10th decile					0.284** (0.021)	0.194** (0.013)
Constant	0.000** (0.000)	0.000** (0.000)	0.000** (0.000)	0.000** (0.000)	0.000** (0.000)	0.000** (0.000)
Observations	3,554,384	3,554,384	2,311,994	2,116,431	2,116,431	2,219,577
Individuals	178,905	178,905	175,232	172,259	172,259	173,858
Emigration events	10,173	10,173	6759	3343	3343	4235

All models control for age (and its square term), marital status, number of children, and having a child under the age of tree, migration background, and previous short migration event. Omitted category in the models—those who did not emigrate

Individual clustered robust standard errors in parentheses

^1^Income including work-related insurance benefits

^2^An indicator of receiving educational allowances

**p < 0.01; *p < 0.05

### Selectivity on Unobserved Characteristics Using School Grades

In general, individuals with higher grades are more prone to emigrate (see Model 2 and Fig. 1) implying that having higher abilities is related to leaving Sweden. This is an important finding, because it applies to all individuals, not only to those attached to the labour market. In addition, as stated before, controlling for school grades reduces the size of most of the education-level coefficients. Model 3 presents the same results for the sample of individuals at the age of 25 or older. While restricting the sample in this way reduces the sample of individuals by just about two per cent, the number of emigration events is reduced significantly leaving 66 and 77 per cent of emigration events of women and men, respectively. This implies, as already stated, that a large share of emigration takes place at an early age. Models 4 and 5 further restrict the sample to individuals attached to the labour market aged 25 and above that had positive income a year before their migration. Similar to the full sample, this restriction further reduces the migration events to 3858 for men and 3343 for women. Again, this clearly shows that emigration occurs many times shortly after graduation among individuals with weak labour force ties.^29^ The coefficients for the school grades in the restricted sample do not differ much from those derived from the full sample (Models 3–6 compared with Model 2) for both gender groups.

### Selectivity on Unobserved Characteristics Using Income Residuals

Following previous studies, we assumed that income residuals from a Mincerian income model capture some of the individuals’ unmeasured abilities. Obviously, the income residuals are a result of the variables chosen to be included in the model and are therefore somewhat arbitrary. However, most previous studies that used income residuals as a measure of unobserved abilities used independent variables similar to those included here.^30^ Appendix 3 presents the Mincerian income models from which the income residuals from work and self-employment are derived. The deciles of income (or income including work-related insurance benefits) residuals are included in Models 5 and 6 in Tables 1 and 2.

Figure 2 presents the yearly emigration rates for men (upper panel) and women (lower panel) by decile of income residuals (and total income residuals—right panel). Starting with men, it is clear that the effect of the income residuals on emigration is U-shaped. Individuals whose income residual is large and negative (those located on the 1st to 4th decile) have a higher emigration rate, while the same is true for individuals with very large positive residuals (9th and 10th deciles). These linkage between negative residuals and emigration tendency imply that individuals who earn significantly less than what they would have expected, based on their observed characteristics, are more prone to leave. The economic literature mainly refers to this as negative self-selection, but it can also represent low job match levels, which result in large negative income residuals. In addition, and as mentioned earlier, individuals might reduce their employment before migration as well as leave in the middle of the year, which might result in lower incomes before migration. At the same time, individuals with high abilities, as measured by large positive income residuals, are also more prone to leave Sweden, which is in line with our expectations regarding positive self-selection. Even when we look at a more inclusive proxy that takes into account transfer payments (Model 6), the results do not change much. The same U-shape pattern is evident among women, but here most emigration is due to negative self-selection in terms of unobserved characteristics. Women with negative income residuals (located at the 1st to the 5th decile) are more prone to leave Sweden than women compensated in accordance with their observed characteristics (6th to 8th deciles).

### Differences in Selectivity Patterns by Migration Destinations

Generally, the Roy model predicts that migrants from Sweden will be positively self-selected. However, sorting (i.e. destination-based differences) in the extent of this positive self-selection are also expected. Therefore, Tables 3 and 4 present the multinomial logit models for men and women, respectively. Model 1 is based on the full sample and Model 2 is estimated for the restricted sample. Each of these models simultaneously estimates the impact of the set of explanatory variables on the four different emigration outcomes—emigration to the Nordic countries, North America and Oceania, Western and Southern European countries, and the rest of the world—compared to the base outcome of staying in Sweden.

**Table 3:** Multinomial logistic regression models for predicting emigration destination from Sweden 1993–2014: Individual men born in Sweden between 1975 and 1978 (relative risk ratio)

	Full sample (1)				Restricted sample**—**age 25+, positive lag income (2)			
	North America, Oceania	Nordic countries	Western Southern Europe	Other	North America, Oceania	Nordic countries	Western Southern Europe	Other
*Educational level—omitted—secondary education*								
Compulsory education nine years or less	2.156** (0.202)	1.087 (0.065)	1.350** (0.123)	1.359* (0.186)	1.683* (0.347)	1.157 (0.132)	1.496* (0.253)	1.029 (0.233)
Post-secondary education less than two	0.597** (0.072)	0.749** (0.062)	1.122 (0.096)	1.267 (0.171)	0.907 (0.192)	0.795 (0.107)	1.187 (0.179)	1.627** (0.295)
Higher education two years or longer	0.647** (0.051)	1.049 (0.058)	1.763** (0.107)	1.520** (0.136)	1.343* (0.176)	1.159 (0.089)	2.499** (0.235)	2.149** (0.258)
Postgraduate	2.043** (0.332)	2.537** (0.480)	4.835** (0.620)	1.508 (0.385)	6.385** (1.368)	2.725** (0.578)	9.893** (1.632)	2.888** (0.859)
CSN^1^	1.236* (0.109)	1.122* (0.065)	0.870* (0.053)	1.062 (0.111)	0.789 (0.136)	0.904 (0.085)	0.724** (0.065)	0.614** (0.103)
Percentile of school grade	1.027** (0.001)	1.003** (0.001)	1.023** (0.001)	1.016** (0.001)	1.021** (0.002)	1.007** (0.001)	1.022** (0.002)	1.013** (0.002)
*Decile of income residual—omitted 1st decile*								
2nd decile					0.585** (0.116)	0.889 (0.099)	0.568** (0.062)	0.620** (0.114)
3rd decile					0.515** (0.095)	0.737** (0.080)	0.502** (0.054)	0.409** (0.075)
4th decile					0.330** (0.068)	0.481** (0.058)	0.315** (0.041)	0.341** (0.064)
5th decile					0.227** (0.053)	0.409** (0.052)	0.230** (0.034)	0.187** (0.040)
6th decile					0.236** (0.051)	0.327** (0.042)	0.215** (0.031)	0.218** (0.044)
7th decile					0.217** (0.047)	0.288** (0.037)	0.220** (0.031)	0.178** (0.037)
8th decile					0.201** (0.044)	0.236** (0.032)	0.222** (0.030)	0.262** (0.048)
9th decile					0.488** (0.085)	0.314** (0.040)	0.301** (0.036)	0.384** (0.065)
10th decile					0.750 (0.121)	0.266** (0.036)	0.506** (0.054)	0.712* (0.109)
Constant	0.000** (0.000)	0.000** (0.000)	0.000** (0.000)	0.000** (0.000)	0.000** (0.000)	0.000** (0.000)	0.000** (0.000)	0.000** (0.000)
Observations	3,763,205				2,277,727			
Individuals	188,159				183,098			

All models control for age (and its square term), marital status, number of children, and having a child under the age of tree, migration background, and previous short migration event

Individual clustered robust standard errors in parentheses

**p < 0.01; *p < 0.05

^1^An indicator of receiving educational allowances

**Table 4:** Multinomial logistic regression models for predicting emigration destination from Sweden 1990–2014: individuals women born in Sweden between 1975 and 1978 (relative risk ratio)

	Full sample (1)				Restricted sample—age 25+, positive lag income (2)			
	North America & Oceania	Nordic countries	Western Southern Europe	Other	North America & Oceania	Nordic countries	Western Southern Europe	Other
*Educational level—omitted—secondary education*								
Compulsory education nine years or less	1.312** (0.123)	0.864* (0.056)	1.084 (0.072)	1.287 (0.182)	0.863 (0.254)	1.264 (0.222)	1.524** (0.233)	1.240 (0.358)
Post-secondary education less than two	0.541** (0.051)	0.716** (0.052)	0.789** (0.043)	0.651** (0.090)	1.057 (0.199)	0.844 (0.126)	1.274* (0.139)	1.006 (0.224)
Higher education two years or longer	0.293** (0.020)	0.855** (0.049)	0.523** (0.021)	0.658** (0.057)	0.862 (0.108)	0.975 (0.090)	1.108 (0.081)	1.137 (0.154)
Postgraduate	0.868 (0.188)	3.481** (0.773)	0.862 (0.150)	0.949 (0.278)	3.677** (1.006)	3.479** (0.888)	2.958** (0.618)	1.766 (0.720)
CSN^1^	1.121 (0.083)	1.059 (0.054)	0.686** (0.031)	0.874 (0.093)	0.658** (0.092)	0.867 (0.087)	0.566** (0.046)	0.682* (0.113)
Percentile of school grade	1.020** (0.001)	1.004** (0.001)	1.021** (0.001)	1.018** (0.002)	1.017** (0.002)	1.009** (0.002)	1.020** (0.001)	1.016** (0.002)
*Decile of income residual—omitted 1st decile*								
2nd decile					0.544** (0.090)	0.744* (0.104)	0.514** (0.052)	0.529** (0.110)
3rd decile					0.468** (0.079)	0.627** (0.089)	0.379** (0.041)	0.616* (0.117)
4th decile					0.381** (0.064)	0.638** (0.087)	0.327** (0.035)	0.377** (0.077)
5th decile					0.198** (0.039)	0.415** (0.063)	0.291** (0.032)	0.299** (0.063)
6th decile					0.151** (0.032)	0.415** (0.061)	0.179** (0.023)	0.328** (0.065)
7th decile					0.149** (0.030)	0.302** (0.046)	0.146** (0.018)	0.177** (0.042)
8th decile					0.143** (0.028)	0.324** (0.047)	0.197** (0.021)	0.197** (0.043)
9th decile					0.127** (0.026)	0.369** (0.052)	0.169** (0.018)	0.217** (0.046)
10th decile					0.223** (0.040)	0.287** (0.047)	0.270** (0.028)	0.394** (0.075)
Constant	0.000** (0.000)	0.000** (0.000)	0.000** (0.000)	0.000** (0.000)	0.000** (0.000)	0.000** (0.000)	0.000** (0.000)	0.000** (0.000)
Observations	3,554,384				2,116,431			
Individuals	178,905				172,259			

All models control for age (and its square term), marital status, number of children, and having a child under the age of tree, migration background, and previous short migration event

Individual clustered robust standard errors in parentheses

^1^An indicator of receiving educational allowances

**p < 0.01; *p < 0.05

**Fig. 3:**
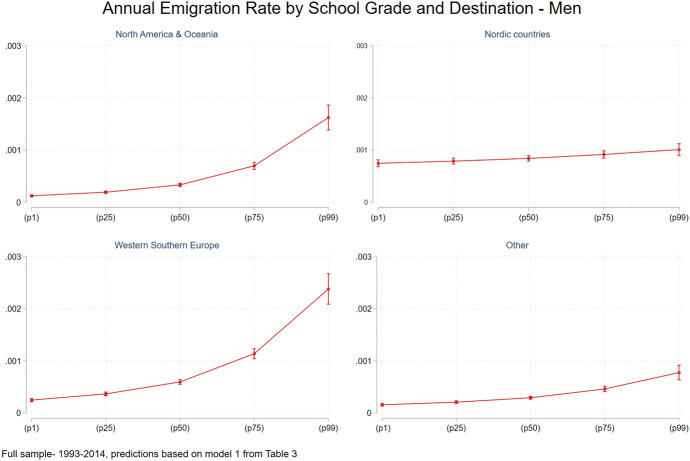
Annual emigration rate by school grade and destination, men

Starting with men, emigrants to the Nordic countries are less positively self-selected compared to emigrants moving to Western and Southern Europe and “other” destinations when looking at higher education that is not postgraduate (Model 1 Table 3). When looking at postgraduate education, the selectivity ranking is emigrants to Western and Southern Europe followed by emigrants to Nordic countries and then emigrants to North America and Oceania. Interestingly, having low levels of education is also associated with a higher risk of emigrating to all destinations except the Nordic countries. Better school performance raises the probability of emigrating to North America and Oceania, Western and Southern Europe, followed by emigrating to “other” and lastly to Nordic countries. Figure 3 presents the annual emigration rate to the four different destinations by school grade, and illustrates, again, that emigration to Nordic countries is less dependent on school grades relative to other destinations, conforming our expectation based on the Roy model.

**Fig. 4:**
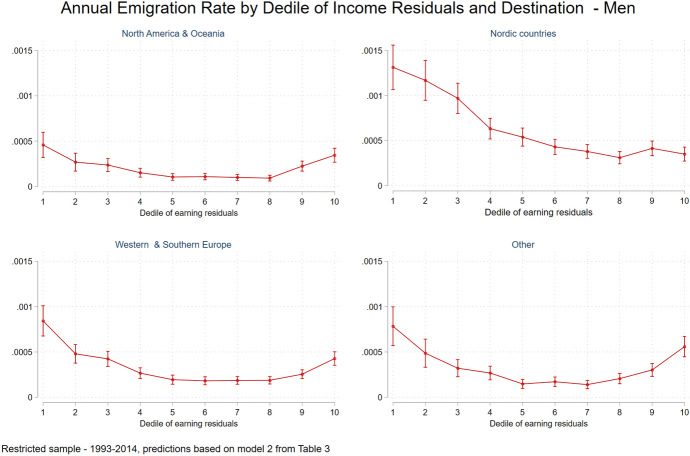
Annual emigration rate by decile of income residuals and destination, men

Model 2 (Table 3) presents the results for the multinomial logit models for the restricted men’s sample that includes decile of income residuals.^31^ As in the logit models, the results of the multinomial logit models show that the effect of income residuals on emigration rates is U-shaped for all destinations, except for the Nordic countries. Figure 4 presents the yearly emigration rates to the four different destinations by decile of income residuals. Again, emigrants to Nordic countries are mainly drawn from the lower part of the incomes residual distribution, implying a negative self-selection in terms of unobserved characteristics, while emigrants to other destinations tend to also be positively self-selected. This U-shape self-selection pattern for all non-Nordic countries suggests that individuals located at the 9th and 10th deciles are more prone to leave relative to those located at the 5th to 8th deciles, and the same is true for those located at the 1st to 4th deciles. Note that the migration rates at the 9th and 10th deciles do not differ much across the three non-Nordic destinations. That being said the overall emigration rates are substantially higher to Nordic and Western and Southern Europe.

Turning now to women, the results of the multinomial logit models with regards to school grades do not change much by gender. Nonetheless, with regards to educational levels, the results are more complex and do not fully match our expectations. The full sample estimation (Table 4, Model 1) suggests that women with non-postgraduate higher education are less prone to emigrate (relative to women with secondary education), and that women with a postgraduate education have much higher relative risk ratios to emigrate to Nordic countries compared to non-Nordic countries. In addition, women with compulsory education have higher risk (relative to women with secondary education) to leave to North America and Oceania. This suggests that other mechanisms explain women’s decision to emigrate from Sweden within the full sample (which also includes younger women). In contrast, when examining the restricted sample (Table 4, Model 2) among women with postgraduate education, we see the following ranking in risk to emigrate: North America and Oceania, Nordic countries, and Western and Southern Europe. However, the effect of higher education that is not postgraduate does not reach statistical significance.

**Fig. 5:**
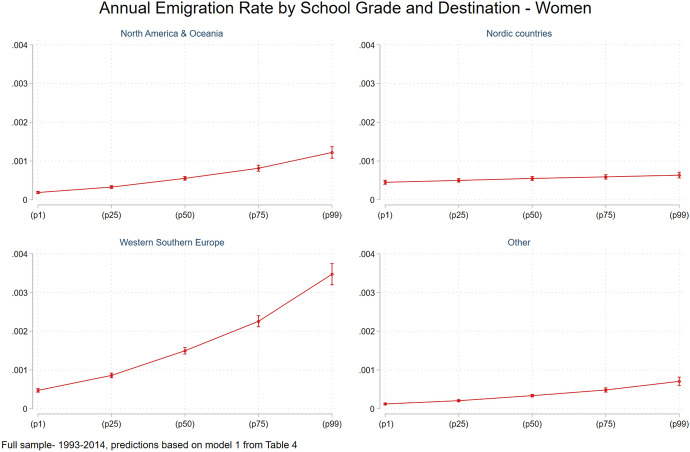
Annual emigration rate by school grade and destination, women

**Fig. 6:**
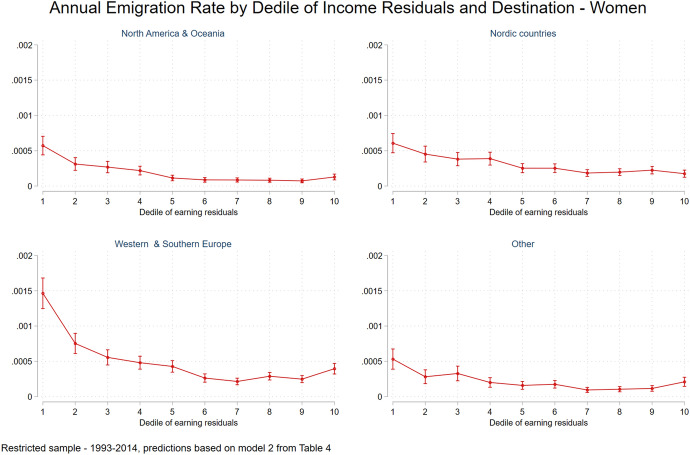
Annual emigration rate by decile of income residuals and destination, women

Similar to men, women's relative risk ratios of emigrating from Sweden based on school grades (Fig. 5) are higher for leaving Sweden to Westerns and Southern Europe, followed by North America and Oceania relative to Nordic countries. Finally, when looking at women’s selectivity levels based on income residuals deciles (Fig. 6), emigrant women to most destination are mainly drawn from the lower part of the residual incomes distribution, while, women emigrating to Westerns and Southern Europe show also a positive pattern of selectivity.

## Conclusions

This study assessed emigrants’ self-selection patterns based on the entire population of potential emigrants in the sending country. This design is unique in migration research and allowed us to test the selectivity hypotheses in a more rigorous way than has been done previously. In addition, it allowed us to examine emigrants’ sorting patterns into different destinations. In doing so, we were able to examine the sorting arguments of Borjas (1987) and Roy (1951) about the effects of differences in rewards between the country of origin and countries of destination on selectivity patterns among emigrants. We examined selectivity levels on both observed and unobserved characteristics. To extend our understanding of the patterns of self-selection in terms of unobserved characteristics, we also used a skills index not directly derived from labour market outcomes namely, school grades while attending compulsory education. Most of the emigration studies restricted their samples of potential emigrants to individuals in their prime working ages who are strongly attached to the labour market (Borjas et al., 2019; Gould & Moav, 2016; Rosso, 2019). In contrast, the current study examined individuals at the ages of 18–39, which enabled us to shed light on selectivity patterns among young emigrants who have not yet positioned themselves in the labour market. The main findings of this paper support, to a large extent, the Roy model. Emigrants from Sweden, a country with low inequality levels, are positively self-selected, while both gender- and destination-based differences exist among them.

Men, on average, tended to be positively self-selected in terms of their characteristics, as measured by educational levels and school grades. As expected, the intensity of positive self-selection among emigrant men to neighbouring Nordic countries is weaker relative to emigrants to other destinations. When measuring unobserved characteristics using income residuals deciles, we found a U-shaped pattern to non-Nordic countries, and higher emigration rates to Nordic countries among individuals with low levels of unobserved characteristics. The fact that emigrants from Sweden to neighbouring Nordic countries, with relatively similar levels of inequality, show a less intense positive self-selection pattern compared to emigrants leaving Sweden to other destinations is also in line with the expectations of the models of Borjas (1987) and Roy (1951). In addition, the higher emigration rate of individuals with large negative income residuals to Nordic countries suggests that individuals that were not compensated based on their observed characteristics were prone to leave. Taking into account the economic crisis that Sweden was going through at the beginning of the 1990s, which raised unemployment levels of low-skilled individuals (Aberg, 2003), and the rising levels of inequality in Sweden during 1990–2014, it is reasonable to assume that many individuals decided to emigrate to other Nordic countries as a low cost employment-searching strategy.^32^

Overall, the U-shaped selection pattern concerning income residuals deciles is in line with Nekby’s (2006) finding concerning the U-shaped pattern of the effect of previous earnings on emigration probabilities of natives from Sweden. Moreover, Nekby (2006) suggested that part of this effect is due to reducing working time prior to migration. In contrast, Borjas et al. (2019) reached different conclusions using earning residuals as a proxy for unobserved abilities: emigrants from Denmark constituted a positively selected subpopulation. Such differences in our findings relative to those of Borjas et al. (2019) could result from several reasons. First and foremost is the sample that we employed compared to that of Borjas et al. (2019), in which we could not (and did not wish to) restrict the sample to full-time employees. We think that Borjas et al., by restricting the sample to full-time employed individuals, are left with a very small share of all migration events. Furthermore, the emigrants they studied were drawn, probably, from the right hand-side tail of the ability distribution. Second, the validity of income residuals as a proxy for abilities both in general and in the specific case of Sweden (where individuals might report emigration retrospectively) is probably low. Finally, our findings about the negative selection of Swedish emigrants to other Nordic countries, can be explained by the unique conditions in each Nordic labour market during the studied period.^33^

Turning to women, we first showed that in line with works on gender-based differences in migration rates (Docquier et al., 2012), Swedish women were more prone to migrate than were men. Second, we found that women were positively self-selected in terms of their school performance and, for women with labour force attachment, this was also true for postgraduate education. Similar to men, we found less favourable selection patterns among women who moved to other Nordic countries relative to other destinations. However, it seems that overall, emigrant women’s levels of selectivity are lower relative to men’s (in terms of education and to some extent also income residuals). This finding can be explained both by additional family considerations shaping women's emigration decision (Borjas et al., 2019; Junge et al., 2014) and by their higher tendency to be tied movers (Bielby & Bielby, 1992; Borjas & Bronars, 1991; Docquier et al., 2012; Mincer, 1978).

Clearly, there are discrepancies in our findings between the effect of school grades and that of the income residuals, both used as proxies of unobserved abilities, on the tendency to emigrate from Sweden. Nonetheless, these finding are in line with Nekby's (2006) finding for Sweden and of other studies that looked at return and onward migration of emigrants (Bijwaard & Wahba, 2014; Dustmann, 2003). As suggested earlier, this might be in part a result of measurement errors that impedes our ability to accurately measure income before emigration (and, of course, the inability to identify full-time employment in our data). However, the findings are in line with expectations. Individuals that have higher unobserved abilities (as measured by school performance and higher residual deciles) are more prone to emigrate. At the same time, individuals that feel that they are not compensated in line with their observed abilities are also prone to leave.

In sum, this paper has made several theoretical and empirical contributions to the migration literature. First, we offered an alternative proxy of unmeasured (and measured) abilities: school performance. This enabled us to show that a large share of the selectivity occurs even before individuals are fully able to display their labour market abilities, suggesting that previous studies have underestimated emigrants’ selectivity levels across developed countries. Second, we established clear differences in the selectivity pattern across destination regions, in line with recent studies that have reached similar conclusions (Borjas et al., 2019; Parey et al., 2017; Rosso, 2019). Finally, our empirical findings regarding the gender-based differences in selectivity levels call for a more in-depth examination of the impacts of gender and family status on emigrants’ selectivity levels.

## Supplementary Information

Below is the link to the electronic supplementary material.

Supplementary file1 (DOCX 55 KB)

## Data Availability

we wish to be exempt from the requirement to make the data available to other researchers as the data are a property of Statistics Sweden.

## Footnotes

1 See for example (Birgier et al., 2016; Chiswick, 1978, 1999; Haberfeld et al., 2020; Parey et al., 2017).

2 Namely, individual (micro-level) decision-making regarding migration are embedded within social contexts of family and network (meso-level) and are based on underlying macro-structural conditions (including economic conditions, migration policies, cultural context, demography and ecology) (Haug, 2008).

3 Specifically, they show that among men, over 70 per cent of the selectivity in pre-migration earnings is due to unobserved characteristics, while among women the equivalent value is 50 per cent.

4 Rosso (2019) showed that among emigrants from Poland, there were significant differences in the patterns of self-selection of individuals who chose Germany versus those who chose the UK. Similarly, in the case of highly skilled emigrants from Germany, individuals who emigrated to less equal countries were more positively selected in terms of their college grades and the quality of the university from which they graduated relative to non-migrants, while emigrants to more equal countries were negatively selected (Parey et al., 2017).

5 For example, by restricting the sample to individuals with strong labour market attachment, Borjas et al. (2019) focused on 25–39 per cent only of migrant men and women, respectively.

6 The common measure of cognitive and non-cognitive abilities in Sweden is based on the Swedish military enlistment tests, which were found to be associated with many life outcomes (Lindqvist & Vestman, 2011). Their drawback for our purposes is that they are limited to young men.

7 The use of grades at an earlier stage in life as a proxy for ability is preferred than grades at a later stage because tracking at individuals at middle and high school is affected by students’ scores at a younger age as well as by their socio-economic background. Therefore, later-stage grades confound ability and background much more than early-stage‏ grades. We used the ninth-grade average grade, which is the earliest grade score available in the data, and is collected before the main educational tracking takes place. Nonetheless, this restricts the number of years for which we can track individuals to 22 years.

8 Clearly, school grade regulates access to higher education, nonetheless the use of the ninth grade average score reduces the direct association with the higher education system. Moreover, while the ninth-year school grades are associated with levels of education, even among individuals with the same level of education, there is a considerable variation in terms of grades. A recent study on internal migration in Sweden and Finland showed selectivity patterns in school grade so that individuals with higher grades were more prone to move to large and medium sized areas (the metropolitan areas) (Eliasson et al., 2020).

9 Other studies that look at the emigration from Sweden during the end of the nineteenth century and the beginning of the 20th century without empirically assessing the Roy model suggest that emigrants were often among the poor, landless, and found in the agricultural sector (Westberg, 2019).

10 https://www.scb.se/en/finding-statistics/statistics-by-subject-area/household-finances/income-and-income-distribution/income-and-tax-statistics/pong/tables-and-graphs/income--consumption-unit-the-entire-country/gini-coefficient/

11 The Nordic countries are Denmark, Finland, Norway, Sweden and Iceland. The three first mentioned borders Sweden.

12 Note that Borjas et al. (2019) did not find differences in their Danish sample between EU and other destinations in terms of emigrant selectivity. This finding led them to suggest that the role of migration policies in shaping selectivity is quite limited.

13 The extent of over-coverage is lower in the case of emigration to other Nordic countries due to shared information between the statistical offices of these countries (Monti et al., 2020).

14 The migration register is based on the residential location of individuals at the time of out-migration. According to Swedish law, an individual who plans to move out of Sweden and to stay abroad for 12 months or more must declare this to the tax authority and to report the destination in question.

15 Administrated by the unit for Human Geography at the University of Gothenburg, it consists of official register data provided by Statistics Sweden from the database Longitudinell Integrationsdatabas för Sjukförsäkrings-och Arbetsmarknadsstudier (LISA, the official name in Swedish; SCB, 2011) and contains data on every individual aged sixteen years or older, registered in Sweden as of December 31 each year.

16 Specifically for Sweden, Nekby (2006) found that migration probabilities are higher for individuals aged 26–35 relative to older individuals.

17 We have full information on more than 98 per cent of individuals for all variables, except lag income. See the “variables” section for an elaboration on our treatment of missing information for lag income.

18 When we restricted the sample to individuals with labour market attachment (e.g. aged 25 and above with positive lagged income), the sample consisted of 183,098 individual men (2,277,727 person-year observations) and 172,259 individual women (2,116,431 person-year observations).

19 Appendices 1 and 2 provide detailed information about the underlying populations of non-emigrants and emigrants.

20 Previous studies used logistics or probit regressions for assessing the probability of leaving, and therefore we followed their methodological approach (Borjas et al., 2019; Gould & Moav, 2016; Rosso, 2019). Nonetheless, the data at hand are derived from a panel. Thus using logistics regression after restructuring the panel by adding time to events essentially results in a discrete-time event history analysis (Allison, 1982; Steele et al., 2004). In this way, each of the independent variables can be interacted with the time of the event for assessing non-proportional hazards. Considering the type of data at hand, this would have been the most appropriate approach rather than the Cox proportional hazards. However, two considerations led us to choose the way we did. First, the result, including time to event, does not differ substantially from the one presented in the paper. Second, as our main interest is in emigration itself and not its timing, we chose to present the more parsimonious‏ analysis.

21 That is, restricting the sample to individuals 25 years and older and with positive lag income from work and self-employment. It should be noted that some individuals tend to reduce their work before emigrating which might bias our results. Most previous studies looked at full-time workers aged 25 and above. As our data do not contain information on working hours, we could not restrict the sample to full-time workers, so we have used the above solution.

22 Individuals that migrated from Sweden between the ages of 16 and 18 were omitted from the sample (416 individuals). It should be noted that we do not have information on whether individuals had migration experience prior to 1990 (from when they were born to the age of 16).

23 Different studies have used various definitions of emigration, ranging from staying abroad for at least five years (Borjas et al., 2019) to at least one year (Gould & Moav, 2016). Appendices 5 and 6 present sensitivity analyses with two different dependent variables: any type of emigration that is longer than 12 months, and a short-term migration (staying less than three years abroad). In these models, individuals can have more than one emigration event. Most of the results do not differ substantially from the results presented in the paper.

24 The other categories were compulsory education 9 years or less, post-secondary education less than 2 years, higher education 2 years or longer, and postgraduate education.

25 Considering the age group of the sample, it might have been more appropriate to measure education by years of schooling. Nonetheless, this variable does not exist in our data. To address the issue of acquiring education, we included an indicator of whether an individual is studying each year (based on information obtained from receiving educational allowances—CSN).

26 The estimated Mincerian income models are presented in appendix 3.

27 Income is in 1996 prices, deflated by the consumer price index.

28 In a sensitivity analysis, we use the income residual and a squared term of the log residual income in the models.

29 Restricting the sample to full-time employees at the ages of 25–54, Borjas et al. (2019) were left with 39 per cent of emigrant men and 25 per cent of women.

30 The variables that are included in the models are marital status, number of children, having a child under the age of 3, age (and its squared term), a sequence of five variables indicating education levels, an indicator of studying, and fixed effects for year and counties of residence. These models explain 26.7 and 30.0 per cent of the variation of lag log yearly incomes from work and self-employment for men and women, respectively.

31 The direction of most effects of the characteristics (educational levels and school grades) does not differ much, yet the effects of most of the education variables are larger in the restricted sample.

32 A similar argument regarding the negative self-selection of those with low abilities, who are more prone to move overseas to seek employment, was offered in the case of young individuals from Poland (Maleszyk, 2021).

33 There were across-country differences during that period within the category of Nordic countries in terms of inequality and employment levels. Parey et al. (2017) presented the following ranked order of income inequality among high-skilled workers in the Nordic countries: Denmark had the lowest level during 1998–2010, followed by Finland, Norway and then Sweden. Also, young individuals (under the age of 25) faced less favourable labour market conditions in Finland and Sweden than in the other Nordic countries during the 1990s (Olofsson & Wadensjö, 2012).

## Code Availability

On demand through the author email‏.
