# Supplementary material for: Movers and Stayers: A Study of Emigration from Sweden 1993–2014
Source: Eur J Popul. 2022 Aug 30;38(5):1033–64. doi: 10.1007/s10680-022-09634-3 (PMC9727015; doi:10.1007/s10680-022-09634-3)
Supplement: Supplementary file 1 — Supplementary file1 (DOCX 55 KB) [file 10680_2022_9634_MOESM1_ESM.docx]

**ONLINE APPENDICES**

| **Appendix 1: Descriptive statistics of full sample individuals: Men and women born in Sweden between 1975-1978** | | | | | | | | | | | | |
| --- | --- | --- | --- | --- | --- | --- | --- | --- | --- | --- | --- | --- |
|  | **Men** | | | | | | **Women** | | | | | |
| Variable | Not migrated | Migrated | North America, Oceania | Nordic countries | Western Southern Europe | Other | Not migrated | Migrated | North America, Oceania | Nordic countries | Western Southern Europe | Other |
| Education: | ***Mean*** | ***Before emigrating*** | | | | | ***Mean*** | ***Before emigrating*** | | | | |
| Compulsory education nine years or less | 0.140 | 0.096 | 0.115 | 0.134 | 0.057 | 0.060 | 0.117 | 0.083 | 0.086 | 0.127 | 0.060 | 0.060 |
| Secondary education | 0.558 | 0.406 | 0.412 | 0.533 | 0.297 | 0.320 | 0.470 | 0.487 | 0.533 | 0.529 | 0.462 | 0.404 |
| Post-secondary education less than two | 0.069 | 0.074 | 0.069 | 0.067 | 0.083 | 0.077 | 0.084 | 0.099 | 0.095 | 0.092 | 0.111 | 0.070 |
| Higher education two years or longer | 0.228 | 0.401 | 0.369 | 0.255 | 0.530 | 0.527 | 0.324 | 0.321 | 0.272 | 0.244 | 0.359 | 0.452 |
| Postgraduate | 0.005 | 0.023 | 0.036 | 0.011 | 0.033 | 0.016 | 0.004 | 0.009 | 0.014 | 0.008 | 0.008 | 0.013 |
| Percentile of high school grade | 42.881 | 56.875 | 61.140 | 46.062 | 65.475 | 59.916 | 54.409 | 64.090 | 64.199 | 58.549 | 66.979 | 66.340 |
|  | 27.911 | 28.724 | 27.667 | 28.597 | 26.360 | 26.681 | 28.096 | 26.169 | 25.166 | 27.819 | 25.085 | 25.443 |
| Married | 0.151 | 0.133 | 0.224 | 0.068 | 0.109 | 0.248 | 0.213 | 0.151 | 0.320 | 0.046 | 0.110 | 0.356 |
| Having a child < 3y | 0.192 | 0.071 | 0.078 | 0.060 | 0.055 | 0.133 | 0.267 | 0.105 | 0.135 | 0.058 | 0.090 | 0.259 |
| N of children | 0.418 | 0.141 | 0.152 | 0.119 | 0.100 | 0.284 | 0.666 | 0.182 | 0.248 | 0.094 | 0.140 | 0.518 |
|  | 0.470 | 0.565 | 0.544 | 0.568 | 0.457 | 0.759 | 0.580 | 0.589 | 0.690 | 0.425 | 0.514 | 0.920 |
| Age | 27.704 | 28.179 | 29.103 | 26.810 | 28.282 | 30.384 | 27.744 | 26.885 | 27.918 | 24.604 | 27.325 | 29.608 |
|  | 0.853 | 4.674 | 4.648 | 4.936 | 4.148 | 3.931 | 0.690 | 4.756 | 4.651 | 4.763 | 4.269 | 4.434 |
| Generation: |  |  |  |  |  |  |  |  |  |  |  |  |
| Both parents are native | 0.857 | 0.760 | 0.772 | 0.734 | 0.776 | 0.777 | 0.858 | 0.759 | 0.749 | 0.765 | 0.763 | 0.745 |
| One parents is native and one immigrants | 0.048 | 0.094 | 0.072 | 0.130 | 0.075 | 0.072 | 0.047 | 0.097 | 0.097 | 0.096 | 0.094 | 0.111 |
| Both parents immigrants | 0.095 | 0.146 | 0.156 | 0.136 | 0.150 | 0.151 | 0.095 | 0.144 | 0.154 | 0.139 | 0.143 | 0.144 |
| Short migration experience | 0.001 | 0.007 | 0.005 | 0.011 | 0.005 | 0.006 | 0.002 | 0.008 | 0.006 | 0.012 | 0.005 | 0.010 |
| Mean number of observation for an individual | 22.397 | 13.137 | 14.069 | 11.763 | 13.249 | 15.323 | 22.471 | 11.847 | 12.885 | 9.567 | 12.293 | 14.543 |
|  | 1.709 | 4.658 | 4.641 | 4.921 | 4.129 | 3.909 | 1.387 | 4.735 | 4.628 | 4.742 | 4.249 | 4.409 |
| ***N*** | 180,092 | 8,067 | 1,414 | 2,940 | 2,602 | 1,111 | 168,732 | 10,173 | 1,715 | 2,846 | 4,630 | 982 |
| ***% emigrates*** | 95.7% | 4.3% |  |  |  |  | 94.3% | 5.7% |  |  |  |  |
| ***% emigrates by destination group*** |  | 100.0% | 17.5% | 36.4% | 32.3% | 13.8% |  | 100.0% | 16.9% | 28.0% | 45.5% | 9.7% |
| For emigrants the descriptive statistics represent the information before emigration from Sweden, for non-emigrants it represents the mean levels at the individual level | | | | | | | | | | | | |

| **Appendix 2: Descriptive statistics of full sample individuals: Men and women born in Sweden between 1975-1978** | | | | | | | | | | | | |
| --- | --- | --- | --- | --- | --- | --- | --- | --- | --- | --- | --- | --- |
|  | **Men** | | | | | | **Women** | | | | | |
| Variable | Not migrated | Migrated | **North America, Oceania** | **Nordic countries** | **Western Southern Europe** | **Other** | Not migrated | Migrated | **North America, Oceania** | **Nordic countries** | **Western Southern Europe** | **Other** |
| Education: | ***Mean*** | ***Before emigrating*** | | | | | ***Mean*** | ***Before emigrating*** | | | | |
| Compulsory education nine years or less | 0.082 | 0.057 | 0.070 | 0.084 | 0.035 | 0.042 | 0.054 | 0.037 | 0.040 | 0.045 | 0.036 | 0.029 |
| Secondary education | 0.523 | 0.312 | 0.310 | 0.433 | 0.218 | 0.282 | 0.412 | 0.319 | 0.384 | 0.327 | 0.289 | 0.327 |
| Post-secondary education less than two | 0.055 | 0.063 | 0.063 | 0.060 | 0.062 | 0.072 | 0.062 | 0.089 | 0.095 | 0.077 | 0.097 | 0.069 |
| Higher education two years or longer | 0.331 | 0.533 | 0.502 | 0.404 | 0.640 | 0.586 | 0.466 | 0.537 | 0.454 | 0.531 | 0.563 | 0.558 |
| Postgraduate | 0.008 | 0.034 | 0.055 | 0.019 | 0.045 | 0.019 | 0.007 | 0.018 | 0.027 | 0.020 | 0.014 | 0.018 |
| Percentile of high school grade | 43.111 | 59.716 | 62.498 | 49.185 | 67.322 | 61.123 | 54.654 | 67.685 | 67.077 | 62.668 | 70.158 | 67.551 |
|  | 27.863 | 28.206 | 27.798 | 28.921 | 25.605 | 26.428 | 27.978 | 25.497 | 25.361 | 27.398 | 24.457 | 25.043 |
| Income |  |  |  |  |  |  |  |  |  |  |  |  |
| Lagged incomes | 2161.1 | 1658.5 | 1841.3 | 1393.8 | 1623.5 | 2022.0 | 1497.4 | 1038.0 | 903.2 | 1191.0 | 1006.5 | 1076.9 |
|  | 925.2 | 1762.4 | 2079.4 | 1275.1 | 1797.5 | 2012.4 | 646.2 | 1157.4 | 1139.4 | 1095.8 | 1177.9 | 1174.3 |
| Ln Lagged incomes | 7.384 | 6.574 | 6.619 | 6.532 | 6.474 | 6.798 | 6.903 | 6.019 | 5.752 | 6.400 | 5.932 | 6.063 |
|  | 0.705 | 1.704 | 1.758 | 1.577 | 1.769 | 1.723 | 0.727 | 1.732 | 1.817 | 1.513 | 1.753 | 1.773 |
| Lagged income & benefits^1^ | 2293.8 | 1846.7 | 2032.2 | 1578.8 | 1828.0 | 2182.1 | 1755.0 | 1295.4 | 1213.4 | 1429.3 | 1253.4 | 1335.4 |
|  | 901.0 | 1679.9 | 2004.9 | 1193.9 | 1697.7 | 1943.9 | 622.8 | 1085.8 | 1098.9 | 988.9 | 1105.3 | 1124.8 |
| Ln Lagged income & benefits^1^ | 7.544 | 7.005 | 7.024 | 6.927 | 6.990 | 7.157 | 7.291 | 6.648 | 6.509 | 6.904 | 6.590 | 6.624 |
|  | 0.596 | 1.313 | 1.428 | 1.243 | 1.300 | 1.335 | 0.559 | 1.351 | 1.426 | 1.128 | 1.363 | 1.469 |
| Information at the mean-lag income residuals | **Mean at the individual** | | | | | | | | | | | |
| 1st decile | 0.052 | 0.182 | 0.186 | 0.161 | 0.209 | 0.165 | 0.078 | 0.211 | 0.254 | 0.154 | 0.227 | 0.194 |
| 2nd decile | 0.053 | 0.157 | 0.155 | 0.150 | 0.174 | 0.136 | 0.069 | 0.145 | 0.156 | 0.123 | 0.154 | 0.133 |
| 3rd decile | 0.079 | 0.142 | 0.130 | 0.153 | 0.145 | 0.125 | 0.074 | 0.125 | 0.123 | 0.128 | 0.125 | 0.120 |
| 4th decile | 0.106 | 0.095 | 0.085 | 0.117 | 0.083 | 0.090 | 0.092 | 0.104 | 0.106 | 0.111 | 0.100 | 0.103 |
| 5th decile | 0.122 | 0.057 | 0.053 | 0.075 | 0.047 | 0.049 | 0.110 | 0.077 | 0.069 | 0.083 | 0.076 | 0.082 |
| 6th decile | 0.127 | 0.053 | 0.046 | 0.064 | 0.046 | 0.054 | 0.121 | 0.059 | 0.054 | 0.074 | 0.052 | 0.062 |
| 7th decile | 0.126 | 0.055 | 0.045 | 0.068 | 0.052 | 0.047 | 0.125 | 0.052 | 0.044 | 0.063 | 0.047 | 0.059 |
| 8th decile | 0.122 | 0.061 | 0.057 | 0.063 | 0.056 | 0.072 | 0.124 | 0.069 | 0.056 | 0.087 | 0.066 | 0.068 |
| 9th decile | 0.113 | 0.080 | 0.096 | 0.074 | 0.070 | 0.093 | 0.116 | 0.075 | 0.057 | 0.097 | 0.071 | 0.075 |
| 10th decile | 0.100 | 0.118 | 0.147 | 0.075 | 0.117 | 0.169 | 0.091 | 0.084 | 0.081 | 0.080 | 0.082 | 0.102 |
|  | ***Mean*** | ***Before emigrating*** | | | | ***Mean*** | ***Before emigrating*** | | | | | |
| Married | 0.230 | 0.160 | 0.267 | 0.103 | 0.120 | 0.130 | 0.313 | 0.188 | 0.367 | 0.089 | 0.127 | 0.330 |
| Having a child < 3y | 0.283 | 0.094 | 0.111 | 0.087 | 0.069 | 0.083 | 0.362 | 0.127 | 0.168 | 0.091 | 0.091 | 0.251 |
| N of children | 0.629 | 0.176 | 0.200 | 0.157 | 0.122 | 0.131 | 0.969 | 0.245 | 0.322 | 0.168 | 0.173 | 0.512 |
|  | 0.690 | 0.594 | 0.599 | 0.592 | 0.483 | 0.410 | 0.813 | 0.661 | 0.734 | 0.559 | 0.558 | 0.910 |
| Age | 31.242 | 29.489 | 29.680 | 29.589 | 28.909 | 30.249 | 31.249 | 28.880 | 28.968 | 29.113 | 28.459 | 29.863 |
|  | 1.008 | 3.323 | 3.449 | 3.261 | 1.713 | 3.292 | 1.011 | 3.253 | 3.403 | 3.207 | 3.062 | 3.517 |
| Generation |  |  |  |  |  |  |  |  |  |  |  |  |
| Both parents are native | 0.858 | 0.786 | 0.795 | 0.767 | 0.794 | 0.796 | 0.859 | 0.786 | 0.780 | 0.801 | 0.784 | 0.779 |
| One parents is native, one immigrants | 0.047 | 0.074 | 0.061 | 0.096 | 0.067 | 0.059 | 0.046 | 0.076 | 0.077 | 0.069 | 0.077 | 0.081 |
| Both parents immigrants | 0.095 | 0.140 | 0.144 | 0.137 | 0.139 | 0.145 | 0.094 | 0.138 | 0.143 | 0.130 | 0.138 | 0.140 |
| ***N*** | 177,698 | 5,400 | 916 | 1,696 | 1,837 | 951 | 167,050 | 5,209 | 881 | 1,104 | 2,496 | 728 |
| ***% emigrates*** | 97.1% | 2.9% |  |  |  |  | 97.0% | 3.0% |  |  |  |  |
| ***% emigrates by destination group*** |  | 100.0% | 17.0% | 31.4% | 34.0% | 17.6% |  | 100.0% | 16.9% | 21.2% | 47.9% | 14.0% |
| For emigrants the descriptive statistics represent the information before emigration from Sweden, for non-emigrants it represents the mean levels at the individual level  ^1^ Income including work-related insurance benefits | | | | | | | | | | | | |

| **Appendix 3: Mincerian Income Regressions by gender: individuals between age 25-39 that earn more than 30 percent of the mean yearly income of the sample** | | | | | | | | |
| --- | --- | --- | --- | --- | --- | --- | --- | --- |
|  | (1) | (2) | (3) | | (1) | (2) | | (3) |
|  | Men | | | | Women | | | |
| VARIABLES | Restricted sample-residuals | RS NON-Mig IND | RS NON-Mig IND | | Restricted sample-residuals | RS Mig IND | | RS NON-Mig IND |
|  |  |  |  | |  |  | |  |
| Age | 0.096** | 0.142** | 0.093** | | 0.126** | 0.185** | | 0.123** |
|  | (0.003) | (0.024) | (0.003) | | (0.003) | (0.022) | | (0.003) |
| Age squared | -0.001** | -0.001** | -0.001** | | -0.001** | -0.002** | | -0.001** |
|  | (0.000) | (0.000) | (0.000) | | (0.000) | (0.000) | | (0.000) |
| Married | 0.060** | 0.097** | 0.059** | | 0.055** | 0.067** | | 0.055** |
|  | (0.001) | (0.011) | (0.001) | | (0.001) | (0.008) | | (0.001) |
| N of children | -0.006** | 0.009 | -0.006** | | -0.105** | -0.117** | | -0.105** |
|  | (0.001) | (0.006) | (0.001) | | (0.001) | (0.005) | | (0.001) |
| Having a child < 3y | 0.004** | 0.034** | 0.002** | | -0.189** | -0.134** | | -0.191** |
|  | (0.001) | (0.009) | (0.001) | | (0.001) | (0.006) | | (0.001) |
| *Educational level - omitted - Secondary education* | | | |  | | |  | |
| Compulsory education nine years | -0.117** | -0.150** | -0.116** | | -0.095** | -0.087** | | -0.095** |
|  | (0.003) | (0.020) | (0.003) | | (0.003) | (0.018) | | (0.003) |
| Post-secondary education less than two | -0.013** | 0.019 | -0.014** | | 0.013** | -0.015 | | 0.015** |
|  | (0.003) | (0.017) | (0.003) | | (0.003) | (0.014) | | (0.003) |
| Higher education two years or longer | 0.076** | 0.107** | 0.075** | | 0.094** | 0.080** | | 0.095** |
|  | (0.001) | (0.010) | (0.001) | | (0.001) | (0.008) | | (0.001) |
| Postgraduate | 0.133** | 0.050** | 0.139** | | 0.241** | 0.158** | | 0.247** |
|  | (0.005) | (0.019) | (0.006) | | (0.006) | (0.021) | | (0.006) |
| CSN^1^ | -0.640** | -0.666** | -0.637** | | -0.613** | -0.618** | | -0.610** |
|  | (0.002) | (0.010) | (0.002) | | (0.002) | (0.009) | | (0.002) |
| Year fixed effects | Yes | Yes | Yes | | Yes | Yes | | Yes |
| Counties fixed effects | Yes | Yes | Yes | | Yes | Yes | | Yes |
| Constant | 5.719** | 4.621** | 5.766** | | 4.921** | 3.751** | | 4.978** |
|  | (0.046) | (0.355) | (0.046) | | (0.052) | (0.330) | | (0.053) |
|  |  |  |  | |  |  | |  |
| Observations | 2,063,211 | 58,779 | 2,004,432 | | 1,811,395 | 58,802 | | 1,752,593 |
| R-squared | 0.267 | 0.339 | 0.264 | | 0.300 | 0.327 | | 0.300 |
| Individual clustered robust standard errors in parentheses, ** p<0.01, * p<0.05  The table reports OLS results for income regression. The dependent variable is measured in 1996 annual gross labour income and/or gross income from business activities (income is in 1996 prices, deflated by the consumer price index).  Coefficient of year and counties fixed effect are not shown  ^1^An indicator of receiving educational allowances | | | | | | | | |

| **Appendix 4: Logistical models for predicting emigration from Sweden 1990-2014: Individuals men and women born in Sweden between 1975-1978 (OR) restricted sample to individuals with 30% lag income** | | | | | | | | |
| --- | --- | --- | --- | --- | --- | --- | --- | --- |
|  | (1) | (2) | (3) | (4) | (1) | (2) | (3) | (4) |
| VARIABLES | Men | | | | Women | | | |
|  | Income | Income & benefits^1^ | Income | Income & benefits^1^ | Income | Income & benefits^1^ | Income | Income & benefits^1^ |
| **Educational level - omitted - Secondary education** | | | | | | | | |
| Compulsory education nine years or less | 1.104 | 1.198* | 1.304** | 1.206* | 1.078 | 1.238* | 1.353* | 1.244* |
|  | (0.106) | (0.109) | (0.127) | (0.109) | (0.135) | (0.130) | (0.172) | (0.131) |
| Post-secondary education less than two | 1.699** | 1.118 | 1.196 | 1.115 | 1.546** | 0.957 | 1.174 | 0.951 |
|  | (0.157) | (0.089) | (0.113) | (0.089) | (0.137) | (0.069) | (0.106) | (0.069) |
| Higher education two years or longer | 3.231** | 1.585** | 1.964** | 1.596** | 1.749** | 0.793** | 1.156* | 0.795** |
|  | (0.146) | (0.078) | (0.106) | (0.079) | (0.086) | (0.039) | (0.066) | (0.039) |
| Postgraduate | 12.190** | 5.229** | 6.363** | 5.157** | 6.766** | 2.569** | 3.599** | 2.541** |
|  | (1.218) | (0.544) | (0.685) | (0.535) | (0.921) | (0.338) | (0.511) | (0.334) |
| CSN^2^ | 0.919 | 1.173** | 0.881 | 1.191** | 0.830* | 1.168** | 0.827** | 1.170** |
|  | (0.083) | (0.068) | (0.076) | (0.069) | (0.061) | (0.059) | (0.060) | (0.059) |
| Percentile of school grade |  | 1.015** | 1.014** | 1.015** |  | 1.018** | 1.015** | 1.018** |
|  |  | (0.001) | (0.001) | (0.001) |  | (0.001) | (0.001) | (0.001) |
| **Decile of income residual - omitted 2^nd^ decile** | | | | | | | | |
| 3rd decile |  |  | 1.117 | 0.781* |  |  | 0.825 | 0.637** |
|  |  |  | (0.296) | (0.085) |  |  | (0.130) | (0.046) |
| 4th decile |  |  | 0.795 | 0.542** |  |  | 0.724* | 0.484** |
|  |  |  | (0.214) | (0.061) |  |  | (0.115) | (0.037) |
| 5th decile |  |  | 0.601 | 0.430** |  |  | 0.549** | 0.407** |
|  |  |  | (0.164) | (0.051) |  |  | (0.089) | (0.033) |
| 6th decile |  |  | 0.551* | 0.379** |  |  | 0.448** | 0.320** |
|  |  |  | (0.150) | (0.045) |  |  | (0.074) | (0.027) |
| 7th decile |  |  | 0.503* | 0.300** |  |  | 0.342** | 0.273** |
|  |  |  | (0.137) | (0.036) |  |  | (0.057) | (0.024) |
| 8th decile |  |  | 0.495** | 0.323** |  |  | 0.403** | 0.262** |
|  |  |  | (0.134) | (0.039) |  |  | (0.065) | (0.022) |
| 9th decile |  |  | 0.746 | 0.412** |  |  | 0.393** | 0.244** |
|  |  |  | (0.201) | (0.048) |  |  | (0.064) | (0.021) |
| 10th decile |  |  | 1.103 | 0.673** |  |  | 0.541** | 0.355** |
|  |  |  | (0.295) | (0.076) |  |  | (0.088) | (0.029) |
| Ln lagged income & benefits residuals |  | 0.919** |  |  |  | 0.940** |  |  |
|  |  | (0.004) |  |  |  | (0.004) |  |  |
| Ln lagged income & benefits residuals squared |  | 1.001** |  |  |  | 1.000** |  |  |
|  |  | (0.000) |  |  |  | (0.000) |  |  |
| Ln lagged income residuals | 1.190** |  |  |  | 0.957 |  |  |  |
|  | (0.050) |  |  |  | (0.050) |  |  |  |
| Ln lagged income residuals squared | 1.574** |  |  |  | 1.598** |  |  |  |
|  | (0.062) |  |  |  | (0.092) |  |  |  |
| Constant | 0.000** | 0.000** | 0.000** | 0.000** | 0.000** | 0.000** | 0.000** | 0.000** |
|  | (0.000) | (0.000) | (0.000) | (0.000) | (0.000) | (0.000) | (0.000) | (0.000) |
|  |  |  |  |  |  |  |  |  |
| Observations | 2,063,211 | 2,204,018 | 2,063,211 | 2,204,018 | 1,811,395 | 2,101,071 | 1,811,395 | 2,101,071 |
| All models control for age (and its square term), marital status, number of children, and having a child under the age of three, migration background, and previous short migration event.  ^1^ Income including work-related insurance benefits  ^2^ An indicator of receiving educational allowances  Individual clustered robust standard errors in parentheses, ** p<0.01, * p<0.05 | | | | | | | | |

| **Appendix 5: Logistical models for predicting emigration from Sweden 1993-2014**  **individual men born in Sweden between 1975-1978, by type of migration (OR)** | | | | | | | | |
| --- | --- | --- | --- | --- | --- | --- | --- | --- |
|  | (1) | (2) | (3) | (4) | (5) | (6) | (7) | (8) |
|  | Full sample | | | | **Restricted sample (age 25+, positive lagged income)** | | | |
| VARIABLES | All type of migration | Short migration | All type of migration | Short migration | All type of migration | Short migration | All type of migration | Short migration |
|  |  |  |  |  |  |  |  |  |
| **Educational level - omitted - Secondary education** | | | | |  | |  | |
| Compulsory education nine years or less | 1.110** | 1.325** | 1.327** | 1.673** | 1.071 | 1.036 | 1.295** | 1.229* |
|  | (0.038) | (0.062) | (0.047) | (0.082) | (0.067) | (0.098) | (0.082) | (0.118) |
| Post-secondary education less than two | 1.558** | 2.840** | 1.165** | 2.034** | 1.779** | 2.478** | 1.365** | 1.929** |
|  | (0.061) | (0.157) | (0.047) | (0.117) | (0.106) | (0.208) | (0.084) | (0.168) |
| Higher education two years or longer | 2.300** | 2.761** | 1.523** | 1.729** | 2.854** | 3.018** | 1.874** | 2.052** |
|  | (0.054) | (0.100) | (0.042) | (0.074) | (0.091) | (0.141) | (0.073) | (0.119) |
| Postgraduate | 8.656** | 9.944** | 4.724** | 5.086** | 13.205** | 13.545** | 7.128** | 7.724** |
|  | (0.566) | (0.962) | (0.327) | (0.518) | (0.979) | (1.429) | (0.573) | (0.894) |
| CSN^1^ | 0.548** | 0.003** | 0.513** | 0.002** | 0.456** | 0.001** | 0.463** | 0.001** |
|  | (0.014) | (0.001) | (0.013) | (0.001) | (0.019) | (0.001) | (0.019) | (0.001) |
| **Decile of imcome residual - omitted 1st decile** | | | | |  | |  | |
| 2nd decile |  |  |  |  | 0.681** | 0.810* | 0.683** | 0.811* |
|  |  |  |  |  | (0.033) | (0.075) | (0.033) | (0.075) |
| 3rd decile |  |  |  |  | 0.556** | 0.564** | 0.560** | 0.569** |
|  |  |  |  |  | (0.026) | (0.049) | (0.026) | (0.050) |
| 4th decile |  |  |  |  | 0.361** | 0.325** | 0.364** | 0.329** |
|  |  |  |  |  | (0.018) | (0.030) | (0.019) | (0.030) |
| 5th decile |  |  |  |  | 0.271** | 0.232** | 0.275** | 0.236** |
|  |  |  |  |  | (0.015) | (0.023) | (0.016) | (0.024) |
| 6th decile |  |  |  |  | 0.216** | 0.171** | 0.216** | 0.172** |
|  |  |  |  |  | (0.013) | (0.018) | (0.013) | (0.018) |
| 7th decile |  |  |  |  | 0.214** | 0.192** | 0.211** | 0.190** |
|  |  |  |  |  | (0.012) | (0.019) | (0.012) | (0.019) |
| 8th decile |  |  |  |  | 0.256** | 0.223** | 0.246** | 0.216** |
|  |  |  |  |  | (0.014) | (0.021) | (0.013) | (0.020) |
| 9th decile |  |  |  |  | 0.360** | 0.291** | 0.336** | 0.276** |
|  |  |  |  |  | (0.018) | (0.025) | (0.017) | (0.024) |
| 10th decile |  |  |  |  | 0.661** | 0.589** | 0.587** | 0.533** |
|  |  |  |  |  | (0.031) | (0.045) | (0.027) | (0.041) |
| Percentile of school grade |  |  | 1.012** | 1.013** |  |  | 1.012** | 1.011** |
|  |  |  | (0.000) | (0.001) |  |  | (0.001) | (0.001) |
| Constant | 0.000** | 0.004** | 0.000** | 0.002** | 0.000** | 0.000** | 0.000** | 0.000** |
|  | (0.000) | (0.002) | (0.000) | (0.001) | (0.000) | (0.000) | (0.000) | (0.000) |
|  |  |  |  |  |  |  |  |  |
| Observations (individual*year) | 3,763,205 | 3,763,205 | 3,763,205 | 3,763,205 | 2,277,727 | 2,277,727 | 2,277,727 | 2,277,727 |
| All models control for age (and its square term), marital status, number of children, and having a child under the age of tree, migration background.  ^1^An indicator of receiving educational allowances  Individual clustered robust standard errors in parentheses, ** p<0.01, * p<0.05 | | | | | | | | |

| **Appendix 6: Logistical models for predicting emigration from Sweden 1993-2014**  **individual women born in Sweden between 1975-1978, by type of migration (OR)** | | | | | | | | |
| --- | --- | --- | --- | --- | --- | --- | --- | --- |
|  | (1) | (2) | (3) | (4) | (7) | (8) | (9) | (10) |
|  | **Full sample** | | | | **Restricted sample (age 25+, positive lagged income)** | | | |
| VARIABLES | All type of migration | Short migration | All type of migration | Short migration | All type of migration | Short migration | All type of migration | Short migration |
| **Educational level - omitted - Secondary education** | | | | | | | | |
| Compulsory education nine years or less | 1.062 | 1.695** | 1.304** | 2.406** | 0.980 | 0.871 | 1.310** | 1.184 |
|  | (0.033) | (0.071) | (0.042) | (0.107) | (0.084) | (0.126) | (0.114) | (0.173) |
| Post-secondary education less than two | 1.253** | 2.898** | 0.943 | 1.935** | 1.859** | 2.701** | 1.393** | 1.957** |
|  | (0.041) | (0.144) | (0.031) | (0.099) | (0.110) | (0.247) | (0.084) | (0.183) |
| Higher education two years or longer | 1.128** | 1.677** | 0.741** | 0.962 | 1.992** | 2.377** | 1.234** | 1.429** |
|  | (0.025) | (0.062) | (0.019) | (0.038) | (0.069) | (0.129) | (0.050) | (0.090) |
| Postgraduate | 3.826** | 5.834** | 1.992** | 2.510** | 8.232** | 9.079** | 3.875** | 4.117** |
|  | (0.336) | (0.767) | (0.178) | (0.334) | (0.845) | (1.366) | (0.414) | (0.646) |
| CSN^1^ | 0.469** | 0.002** | 0.429** | 0.002** | 0.423** | 0.003** | 0.431** | 0.003** |
|  | (0.010) | (0.000) | (0.009) | (0.000) | (0.017) | (0.002) | (0.017) | (0.002) |
| **Decile of income residual - omitted 1st decile** |  |  |  |  |  |  |  |  |
| 2nd decile |  |  |  |  | 0.625** | 0.729** | 0.625** | 0.729** |
|  |  |  |  |  | (0.030) | (0.067) | (0.031) | (0.067) |
| 3rd decile |  |  |  |  | 0.470** | 0.432** | 0.469** | 0.430** |
|  |  |  |  |  | (0.024) | (0.044) | (0.024) | (0.044) |
| 4th decile |  |  |  |  | 0.413** | 0.351** | 0.410** | 0.349** |
|  |  |  |  |  | (0.021) | (0.034) | (0.021) | (0.034) |
| 5th decile |  |  |  |  | 0.300** | 0.265** | 0.301** | 0.266** |
|  |  |  |  |  | (0.016) | (0.025) | (0.016) | (0.026) |
| 6th decile |  |  |  |  | 0.237** | 0.187** | 0.240** | 0.190** |
|  |  |  |  |  | (0.013) | (0.019) | (0.014) | (0.019) |
| 7th decile |  |  |  |  | 0.192** | 0.157** | 0.191** | 0.156** |
|  |  |  |  |  | (0.011) | (0.016) | (0.011) | (0.016) |
| 8th decile |  |  |  |  | 0.232** | 0.190** | 0.221** | 0.182** |
|  |  |  |  |  | (0.012) | (0.017) | (0.012) | (0.017) |
| 9th decile |  |  |  |  | 0.240** | 0.189** | 0.219** | 0.173** |
|  |  |  |  |  | (0.013) | (0.017) | (0.012) | (0.016) |
| 10th decile |  |  |  |  | 0.382** | 0.313** | 0.323** | 0.265** |
|  |  |  |  |  | (0.020) | (0.027) | (0.017) | (0.023) |
| Percentile of school grade |  |  | 1.014** | 1.019** |  |  | 1.017** | 1.017** |
|  |  |  | (0.000) | (0.001) |  |  | (0.001) | (0.001) |
| Constant | 0.000** | 2.437* | 0.000** | 0.509 | 0.000** | 0.003** | 0.000** | 0.001** |
|  | (0.000) | (0.932) | (0.000) | (0.195) | (0.000) | (0.005) | (0.000) | (0.001) |
|  |  |  |  |  |  |  |  |  |
| Observations (individual*year) | 3,554,384 | 3,554,384 | 3,554,384 | 3,554,384 | 2,116,431 | 2,116,431 | 2,116,431 | 2,116,431 |
| All models control for age (and its square term), marital status, number of children, and having a child under the age of tree, migration background.  ^1^An indicator of receiving educational allowances  Individual clustered robust standard errors in parentheses, ** p<0.01, * p<0.05 | | | | | | | | |

| **Appendix 7: Logistical models for predicting emigration from Sweden 1993-2014**  **Individuals men and women born in Sweden between 1975-1978 (OR)** | | | | | | | | | | | | |
| --- | --- | --- | --- | --- | --- | --- | --- | --- | --- | --- | --- | --- |
|  | (1) | (2) | (3) | (4) | (5) | (6) | (1) | (2) | (3) | (4) | (5) | (6) |
|  | **Men** | | | | | | **Women** | | | | | |
| VARIABLES | **Full sample** | | **Restricted sample** | | | | **Full sample** | | **Restricted sample** | | | |
|  |  |  | Age 25+ | Age 25+ and lagged income | | Age 25+ and lagged income & benefits^1^ |  |  | Age 25+ | Age 25+ and lagged income | | Age 25+ and lagged income & benefits^1^ |
| *Educational level -  omitted - Secondary education* |  |  |  |  |  |  |  |  |  |  |  |  |
|  | (0.053) | (0.054) | (0.060) | (0.075) | (0.072) | (0.067) | (0.047) | (0.047) | (0.056) | (0.080) | (0.076) | (0.064) |
| Compulsory education nine years or less | 1.112** | 1.418** | 1.455** | 1.483** | 1.293** | 1.118 | 0.893** | 1.148** | 1.352** | 1.480** | 1.293* | 1.068 |
|  | (0.045) | (0.060) | (0.080) | (0.119) | (0.104) | (0.086) | (0.034) | (0.046) | (0.079) | (0.148) | (0.130) | (0.092) |
| Post-secondary education less than two | 1.201** | 0.840** | 1.020 | 1.117 | 1.013 | 1.055 | 0.920* | 0.683** | 0.848** | 1.115 | 1.082 | 0.943 |
|  | (0.058) | (0.042) | (0.060) | (0.090) | (0.081) | (0.074) | (0.034) | (0.026) | (0.041) | (0.084) | (0.081) | (0.059) |
| Higher education two years or longer | 2.081** | 1.256** | 1.204** | 1.828** | 1.661** | 1.484** | 0.869** | 0.561** | 0.522** | 1.087 | 1.036 | 0.775** |
|  | (0.058) | (0.041) | (0.044) | (0.086) | (0.079) | (0.067) | (0.022) | (0.016) | (0.017) | (0.053) | (0.051) | (0.034) |
| Postgraduate | 6.494** | 3.096** | 2.862** | 5.073** | 5.133** | 4.510** | 2.451** | 1.235 | 1.081 | 3.130** | 3.046** | 2.204** |
|  | (0.523) | (0.261) | (0.246) | (0.510) | (0.517) | (0.444) | (0.263) | (0.135) | (0.119) | (0.414) | (0.405) | (0.276) |
| CSN^2^ | 1.059 | 1.020 | 1.191** | 1.238** | 0.781** | 1.082 | 0.922** | 0.874** | 1.025 | 1.112* | 0.665** | 1.028 |
|  | (0.037) | (0.036) | (0.049) | (0.064) | (0.045) | (0.053) | (0.026) | (0.025) | (0.036) | (0.054) | (0.036) | (0.045) |
| Year group - omitted 2001-2005 |  |  |  |  |  |  |  |  |  |  |  |  |
| 1991-1995 | 0.538** | 0.520** |  |  |  |  | 0.577** | 0.554** |  |  |  |  |
|  | (0.063) | (0.061) |  |  |  |  | (0.049) | (0.047) |  |  |  |  |
| 1996-2000 | 0.956 | 0.962 | 0.824 | 0.820 | 0.791 | 0.844 | 0.933 | 0.934 | 0.926 | 1.097 | 1.084 | 0.956 |
|  | (0.046) | (0.047) | (0.084) | (0.103) | (0.100) | (0.092) | (0.037) | (0.038) | (0.076) | (0.117) | (0.115) | (0.087) |
| 2006-2010 | 1.131** | 1.102* | 1.020 | 1.051 | 1.070 | 1.031 | 0.994 | 0.980 | 0.910* | 0.918 | 0.922 | 0.902 |
|  | (0.047) | (0.045) | (0.047) | (0.062) | (0.064) | (0.057) | (0.039) | (0.038) | (0.039) | (0.057) | (0.058) | (0.051) |
| 2011-2014 | 1.216* | 1.197* | 1.195* | 1.292* | 1.310** | 1.226* | 0.985 | 1.014 | 1.016 | 1.104 | 1.109 | 1.077 |
|  | (0.099) | (0.097) | (0.096) | (0.131) | (0.133) | (0.120) | (0.082) | (0.083) | (0.083) | (0.130) | (0.130) | (0.116) |
| Percentile of school grade |  | 1.015** | 1.018** | 1.015** | 1.015** | 1.016** |  | 1.015** | 1.020** | 1.015** | 1.016** | 1.018** |
|  |  | (0.001) | (0.001) | (0.001) | (0.001) | (0.001) |  | (0.000) | (0.001) | (0.001) | (0.001) | (0.001) |
| Decile of income residual - omitted 1st decile | | |  |  |  |  |  |  |  |  |  |  |
| 2nd decile |  |  |  |  | 0.679** | 0.753** |  |  |  |  | 0.569** | 0.688** |
|  |  |  |  |  | (0.046) | (0.053) |  |  |  |  | (0.039) | (0.042) |
| 3rd decile |  |  |  |  | 0.562** | 0.506** |  |  |  |  | 0.473** | 0.371** |
|  |  |  |  |  | (0.037) | (0.034) |  |  |  |  | (0.033) | (0.023) |
| 4th decile |  |  |  |  | 0.373** | 0.343** |  |  |  |  | 0.407** | 0.280** |
|  |  |  |  |  | (0.028) | (0.025) |  |  |  |  | (0.029) | (0.018) |
| 5th decile |  |  |  |  | 0.276** | 0.266** |  |  |  |  | 0.299** | 0.231** |
|  |  |  |  |  | (0.023) | (0.021) |  |  |  |  | (0.023) | (0.016) |
| 6th decile |  |  |  |  | 0.252** | 0.232** |  |  |  |  | 0.240** | 0.180** |
|  |  |  |  |  | (0.020) | (0.018) |  |  |  |  | (0.019) | (0.013) |
| 7th decile |  |  |  |  | 0.230** | 0.183** |  |  |  |  | 0.182** | 0.152** |
|  |  |  |  |  | (0.018) | (0.015) |  |  |  |  | (0.015) | (0.011) |
| 8th decile |  |  |  |  | 0.227** | 0.197** |  |  |  |  | 0.212** | 0.144** |
|  |  |  |  |  | (0.018) | (0.015) |  |  |  |  | (0.016) | (0.010) |
| 9th decile |  |  |  |  | 0.345** | 0.251** |  |  |  |  | 0.206** | 0.133** |
|  |  |  |  |  | (0.024) | (0.018) |  |  |  |  | (0.015) | (0.010) |
| 10th decile |  |  |  |  | 0.511** | 0.411** |  |  |  |  | 0.284** | 0.193** |
|  |  |  |  |  | (0.033) | (0.027) |  |  |  |  | (0.021) | (0.013) |
| Constant | 0.000** | 0.000** | 0.000** | 0.000** | 0.000** | 0.000** | 0.000** | 0.000** | 0.000** | 0.000** | 0.000** | 0.000** |
|  | (0.000) | (0.000) | (0.000) | (0.000) | (0.000) | (0.000) | (0.000) | (0.000) | (0.000) | (0.000) | (0.000) | (0.000) |
|  |  |  |  |  |  |  |  |  |  |  |  |  |
| Observations (individual*year) | 3,763,205 | 3,763,205 | 2,452,999 | 2,277,727 | 2,277,727 | 2,337,644 | 3,554,384 | 3,554,384 | 2,311,994 | 2,116,431 | 2,116,431 | 2,219,577 |
| All models control for age (and its square term), marital status, number of children, and having a child under the age of three, migration background, and previous short migration event.  ^1^ Income including work-related insurance benefits  ^2^ An indicator of receiving educational allowances  Individual clustered robust standard errors in parentheses, ** p<0.01, * p<0.05 | | | | | | | | | | | | |

| **Appendix 8: Destination countries grouped into 4 categories** | | | |
| --- | --- | --- | --- |
| **Nordic** | **Other** | | |
| Denmark | Afghanistan | India | Thailand |
| Finland | Africa | Iraq | Tunisia |
| Iceland | Algeria | Japan | Turkey |
| Norway | Argentina | Korea, south | Uganda |
| **Western & Sothern Europe** | Bangladesh | Lebanon | Unknown |
| Austria | Bolivia | Lithuania | Vietnam |
| Belgium | Bosnia-Herzegovina | Macedonia | Yugoslavia |
| France | Brazil | Middle America |  |
| Germany | Bulgaria | Middle east |  |
| Great Britain | Chile | Morocco |  |
| Greece | China | Pakistan |  |
| Ireland | Colombia | Peru |  |
| Italy | Croatia | Philippines |  |
| Netherlands | Czechoslovakia | Poland |  |
| Portugal | East Asia | Romania |  |
| Spain | Egypt | Russia |  |
| Switzerland | Eritrea | Slovenia |  |
| **North America and Oceania** | Estonia | Somalia |  |
| Australia | Ethiopia | South America |  |
| Canada | Europe, small | Soviet union |  |
| New Zealand | Gambia | Sri Lanka |  |
| USA | Hungary | Syria |  |
